# Long-term mortality trends in patients with concurrent hernia and diabetes in the United States

**DOI:** 10.3389/fendo.2026.1723074

**Published:** 2026-02-19

**Authors:** Xiaoyang Shi, Xiao Yu, Yu-Jun Xiong, Tian Lv, Xiaoyong Si

**Affiliations:** 1 Department of Hernia and Abdominal Wall Surgery, Peking University People’s Hospital, Beijing, China; 2 Department of Otolaryngology-Head and Neck Surgery, Shanghai Sixth People’s Hospital Affiliated to Shanghai Jiao Tong University School of Medicine, Shanghai, China; 3 Department of Gastroenterology, Beijing Hospital, National Center of Gerontology, Institute of Geriatric Medicine, Chinese Academy of Medical Sciences, Beijing, China; 4 Department of Neurology, Zhuji Affiliated Hospital of Wenzhou Medical University, Zhuji, China; 5 Department of Science and Education, Zhuji Affiliated Hospital of Wenzhou Medical University, Zhuji, China

**Keywords:** age-adjusted mortality rate, annual percent change, diabetes mellitus, hernia, mortality trends

## Abstract

**Background:**

The coexistence of hernia and diabetes mellitus presents substantial clinical challenges due to diabetes-related impairment of wound healing, immune response, and vascular function. Despite this established pathophysiological relationship, national mortality trends for patients with both conditions remain inadequately characterized.

**Methods:**

This population-based study analyzed U.S. mortality data from 1999-2023. We included decedents aged ≥25 years with both hernia (ICD-10 K40–K46) and diabetes mellitus (E10–E14) listed as contributing causes of death. Age-adjusted mortality rates were calculated using the 2000 U.S. standard population. Joinpoint regression analyzed temporal trends stratified by demographic and geographic factors.

**Results:**

From 1999 to 2023, 7,128 U.S. deaths involved both hernia and diabetes. Overall age-adjusted mortality rates showed no significant long-term change, with a modest decline before 2019 followed by a sharp increase thereafter. Mortality rose more prominently among males, older adults, and individuals aged 65 years and above. Females and younger age groups experienced earlier declines but demonstrated clear reversals in recent years. Regional analyses revealed sustained declines in the Midwest, relative stability in the Northeast, and increasing mortality in the South and West. Racial and ethnic disparities persisted, with recent upward trends observed among Non-Hispanic White, Hispanic, and Non-Hispanic Black populations. Metropolitan areas showed greater mortality reductions than nonmetropolitan areas.

**Conclusions:**

Mortality involving both hernia and diabetes in the United States has remained largely stable over the past two decades but has increased markedly in recent years. Substantial disparities by sex, age, race, region, and urbanization persist. These findings highlight the need for improved perioperative risk management, equitable access to timely hernia repair, and integrated diabetes care, particularly for older adults and underserved populations.

## 1 Introduction

Hernias of the abdominal wall—such as inguinal, femoral, umbilical, and ventral hernias—are among the most common surgical conditions globally. Although age-standardized incidence rates have declined, the absolute number of people affected continues to rise, contributing to a growing disease burden (1). Recent global estimates indicate tens of millions of prevalent cases, with increasing incidents of hernia-related disability and non-negligible mortality, particularly in complicated cases (2). Concurrently, diabetes mellitus represents a major and expanding public health challenge worldwide. Current reports suggest that hundreds of millions of adults live with diabetes, which has been responsible for more than one million annual deaths in recent years, with rising prevalence and mortality noted especially in middle- and low-income countries (3).

The clinical interplay between hernias and diabetes is of considerable importance, as diabetes impairs wound healing, immune function, and microvascular integrity—factors critical to managing hernia complications and repair outcomes (4). Previous studies have consistently shown that diabetic patients undergoing abdominal wall or hernia repair experience higher rates of wound complications, surgical site infections, extended hospital stays, and recurrence (5). For instance, Soare et al. identified diabetes and obesity as independent risk factors for wound-related adverse events following ventral hernia repair (6). Similarly, Messer et al. observed that diabetes was associated with increased wound morbidity in complex abdominal wall reconstruction, although no specific HbA1c threshold could be established to preclude surgery (7). In an ambulatory surgery cohort, Shanahan et al. demonstrated that preoperative hyperglycemia exceeding 180 mg/dL significantly increased the probability of adverse events within 14 days, yielding an adjusted odds ratio of about 1.5 (8).

Despite these findings, existing research remains largely confined to short-term or perioperative outcomes, with a scarcity of population-level studies on long-term mortality. To address this gap, we conducted a retrospective analysis of U.S. mortality data from 1999 to 2023 within CDC WONDER, focusing on adults aged ≥25 years with both hernia (ICD-10 K40–K46) and diabetes mellitus (E10–E14) listed as causes of death, without assuming hernia or diabetes mellitus only as the proximate cause of death. This study aims to characterize long-term mortality trends in this population and explore demographic and geographic variations to inform clinical and public health strategies.

## 2 Materials and methods

### 2.1 Study design and inclusion criteria

This retrospective, population-based study analyzed long-term mortality patterns from 1999 to 2023 in a cohort defined by the concurrent mention of hernia and diabetes on death causes. The analysis relied on a complete national mortality database (9). Inclusion required individuals to be aged 25 years or above, with both hernia (ICD-10 K40-K46) and diabetes mellitus (ICD-10 E10-E14) as contributing causes, regardless of underlying cause of death. For adults aged 18 years and older, CDC WONDER reports age categories beginning at 25–34 years. Accordingly, analyses were restricted to individuals aged ≥25 years to ensure consistency with the available age strata. Records were excluded if they lacked complete data on core demographic variables (age, sex, race) or fell outside the registry’s coverage (10). ICD-10 coding practices and reporting behavior may vary over time, which should be considered when interpreting long-term trends.

### 2.2 Data extraction and subgroup analyses

Data on total deaths, population sizes, and key demographic variables were obtained. Mortality rates were analyzed across specific age cohorts (25–34, 35-44, 45–54, 55–64, 65-74, 75-84, and ≥85 years), sex, racial groups (Hispanic, non-Hispanic White, non-Hispanic Black, and non-Hispanic Other), and U.S. census regions (Northeast, Midwest, South, and West). Urban-rural designations were assigned according to the National Center for Health Statistics Urban-Rural Classification Scheme, where metropolitan areas encompass large central, large fringe, medium, and small metropolitan counties, and nonmetropolitan areas include micropolitan and noncore counties (see **Supplementary Tables 1–3**). This multi-level stratification enabled a detailed evaluation of mortality disparities by demographic, regional, and urban-rural characteristics (11).

### 2.3 Statistical analysis

This study calculated crude mortality rates as the number of annual deaths involving both hernia and diabetes per the corresponding U.S. population estimate. Age-adjusted mortality rates (AAMRs) were derived by direct standardization against the year 2000 U.S. standard population. Temporal trends in AAMRs were analyzed using JoinPoint Regression Software (version 5.4.0.1; National Cancer Institute), which allows for the identification of statistically significant changes in mortality slopes over time and provides a flexible framework for detecting trend inflection points across demographic subgroups. For each identified trend segment, the annual percent change (APC) was computed, and the average annual percent change (AAPC) over the entire period was determined as a summary measure. The statistical significance of these trends was evaluated using a t-test, with an alpha level of 0.05, to determine whether the APC or AAPC significantly differed from zero. This methodology enabled a detailed quantification of national mortality trends, stratified by demographic and geographic subgroups. To preserve data integrity, mortality rates, AAMRs, and APC/AAPC values are reported as blank or “NA” in figures and supplementary tables when state- or year-specific data were insufficient to ensure reliable estimation.

## 3 Results

### 3.1 Overall characteristics

Between 1999 and 2023, a total of 7,128 deaths in the United States were documented with both hernia and diabetes listed on the death certificate (**Table 1**). Overall age-adjusted mortality rates showed temporal fluctuations but no statistically significant long-term change, increasing slightly from 0.14 to 0.16 per 100,000 population over the study period, with a non-significant average annual percent change of 0.86% (P > 0.05). Joinpoint regression identified two distinct temporal phases in overall mortality trends. Mortality declined significantly from 1999 to 2019, with an annual percent change of −1.50% (95% CI −2.31 to −0.69). This favorable trend reversed after 2019, followed by a pronounced and statistically significant increase in mortality during 2019–2023, with an annual percent change of 13.55% (95% CI 4.78 to 23.05).

**Table 1:** Trends in mortality and age-adjusted mortality rates for hernia with concurrent diabetes mellitus in 1999 and 2023.

Characteristics	Deaths			AAMR		
	1999	2023	Percent change	1999	2023	AAPC (95% CI)
Overall	252	425	68.65	0.14 (0.12 to 0.15)	0.16 (0.14 to 0.17)	0.86 (-0.56 to 2.30)
Sex						
Female	174	231	32.76	0.16 (0.14 to 0.19)	0.15 (0.13 to 0.17)	-0.26 (-1.68 to 1.18)
Male	78	194	148.72	0.11 (0.09 to 0.14)	0.15 (0.13 to 0.17)	1.28 (-0.28 to 2.86)
Census Region						
Northeast	51	63	23.53	0.12 (0.09 to 0.16)	0.11 (0.08 to 0.14)	-0.09 (-1.15 to 0.99)
Midwest	72	80	11.11	0.17 (0.13 to 0.21)	0.13 (0.10 to 0.16)	-1.87 (-2.67 to -1.07)*
South	79	193	144.3	0.13 (0.10 to 0.16)	0.18 (0.15 to 0.20)	0.93 (-0.06 to 1.94)
West	50	89	78	0.13 (0.09 to 0.17)	0.15 (0.12 to 0.19)	1.56 (0.50 to 2.64)*
Races						
Hispanic	13	59	353.85	NA (0.08 to 0.28)	0.21 (0.16 to 0.27)	
NH Black	26	67	157.69	0.18 (0.12 to 0.26)	0.23 (0.17 to 0.29)	-0.53 (-1.72 to 0.68)
NH White	205	284	38.54	0.13 (0.11 to 0.15)	0.14 (0.13 to 0.16)	0.51 (-0.78 to 1.81)
NH Other		14	NA	NA (NA to NA)	NA (0.03 to 0.11)	
Urbanization						
Metropolitan	193	251	30.05	0.13 (0.11 to 0.15)	0.10 (0.09 to 0.11)	-1.33 (-2.01 to -0.64)*
Nonmetropolitan	59	73	23.73	0.17 (0.13 to 0.22)	0.17 (0.13 to 0.22)	-0.72 (-1.83 to 0.40)
Age groups						
25–34 years			NA	NA (NA to NA)	NA (NA to NA)	
35–44 years			NA	NA (NA to NA)	NA (NA to NA)	
45–54 years		29	NA	NA (NA to NA)	0.07 (0.05 to 0.10)	
55–64 years	23	72	213.04	0.10 (0.06 to 0.15)	0.17 (0.13 to 0.22)	0.62 (-0.46 to 1.72)
65–74 years	58	103	77.59	0.31 (0.24 to 0.41)	0.30 (0.24 to 0.35)	0.54 (-0.79 to 1.88)
75–84 years	71	118	66.2	0.58 (0.45 to 0.73)	0.64 (0.53 to 0.76)	-0.19 (-3.96 to 3.73)
85+ years	89	92	3.37	2.14 (1.72 to 2.64)	1.49 (1.20 to 1.82)	-1.35 (-3.29 to 0.62)

*Indicates statistically significant AAPC.

#Urbanization-specific AAMR for 2023 is based on 2020 data. AAPC was calculated for 1999–2023.

##Age-group AAMR and APC are calculated using crude rates.

Age adjusted mortality rate (AAMR), confidence interval (CI), average annual percentage change (AAPC), non-Hispanic (NH).

### 3.2 Sex-stratified analyses

**Figure 1:**
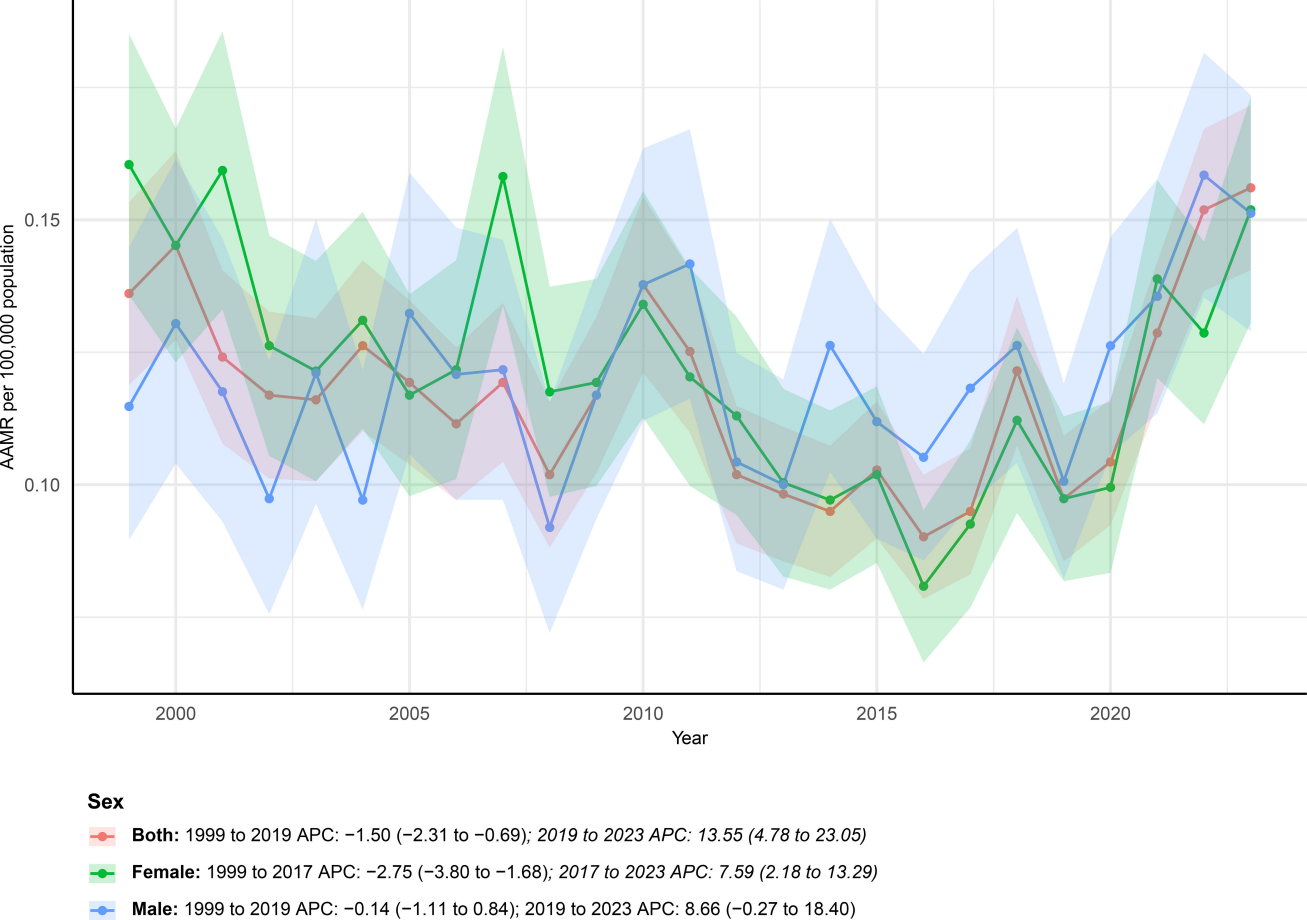
Age-adjusted mortality rates (AAMRs) of mortality involving hernia and diabetes among U.S. patients stratified by sex from 1999–2023.

Sex-stratified analyses revealed divergent mortality trajectories between males and females (**Figure 1**). Females accounted for 4,144 deaths, whereas 2,984 deaths occurred among males, as shown in **Figure 1**. Among males, the AAMR increased from 0.11 (95% CI 0.09–0.14) in 1999 to 0.15 (95% CI 0.13–0.17) per 100,000 population in 2023, corresponding to a non-significant overall upward trend with an AAPC of 1.28% (95% CI −0.28 to 2.86). Joinpoint regression identified two temporal phases for males, including a relatively stable period from 1999 to 2019 with an APC of −0.14% (95% CI −1.11 to 0.84), followed by a marked increase between 2019 and 2023 with an APC of 8.66% (95% CI −0.27 to 18.40).

In contrast, females exhibited a modest overall decline in mortality, with the AAMR decreasing slightly from 0.16 (95% CI 0.14–0.19) in 1999 to 0.15 (95% CI 0.13–0.17) per 100,000 in 2023 and an AAPC of −0.26% (95% CI −1.68 to 1.18). Trend analysis revealed a significant reduction from 1999 to 2017 with an APC of −2.75% (95% CI −3.80 to −1.68), followed by a pronounced reversal characterized by a rapid increase in mortality during 2017–2023 with an APC of 7.59% (95% CI 2.18 to 13.29).

### 3.3 Age-stratified analyses

**Figure 2:**
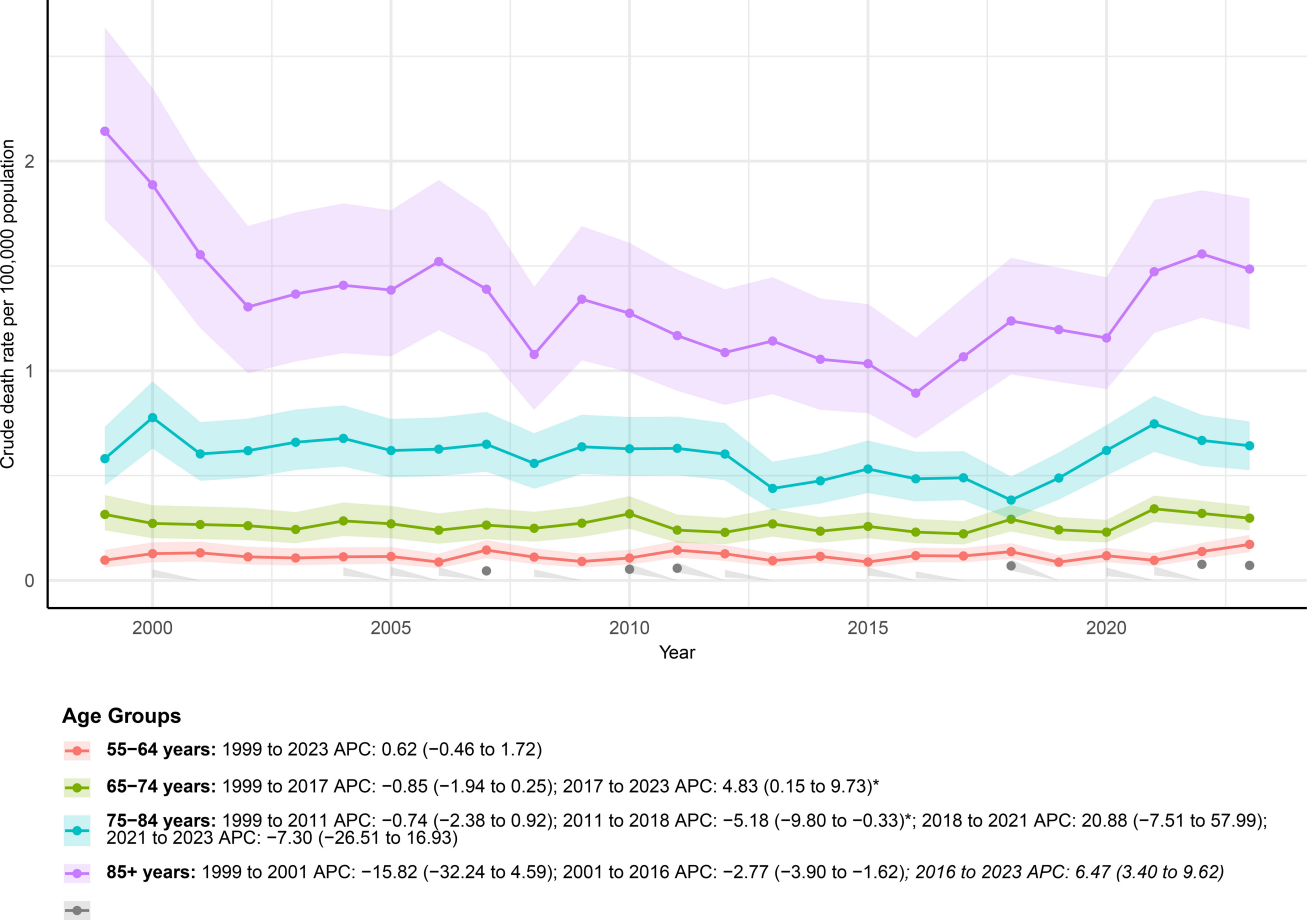
AAMRs of mortality involving hernia and diabetes among U.S. patients stratified by age group from 1999–2023. Age-specific trend lines are not shown for individuals aged 25–54 years because of missing or suppressed data in CDC WONDER.

Age-stratified analyses demonstrated pronounced heterogeneity in mortality trends, with clearer temporal reversals observed in older age groups. Mortality patterns differed markedly across age groups, as illustrated in **Figure 2**. Due to incomplete data availability for individuals aged 25–54 years, mortality trends for these age groups were not displayed in **Figure 2** and were not summarized in **Table 1**. Among adults aged 55–64 years, the age-adjusted mortality rate increased from 0.10 (95% CI 0.06–0.15) per 100,000 population in 1999 to 0.17 (95% CI 0.13–0.22) in 2023, corresponding to a modest and statistically non-significant upward trend over the study period. Joinpoint analysis indicated a generally stable pattern from 1999 to 2023, with no distinct inflection points identified.

For individuals aged 65–74 years, mortality declined modestly between 1999 and 2017, followed by a significant upward reversal after 2017. Specifically, the trend shifted from a decreasing phase during 1999–2017 to a marked increase between 2017 and 2023, during which mortality rose rapidly. A similar but more volatile pattern was observed among those aged 75–84 years. Mortality decreased gradually from 1999 to 2011, followed by a steeper decline through 2018. This downward trend was interrupted by a transient surge during 2018–2021, after which mortality declined again in the most recent period.

Among the oldest age group aged 85 years and older, mortality exhibited pronounced long-term variability. A sharp decline was observed in the early years of the study, followed by a sustained reduction through 2016. From 2016 onward, mortality increased significantly through 2023, although the overall long-term trend remained characterized by substantial fluctuations rather than a consistent directional change.

### 3.4 Regional-stratified analyses

**Figure 3:**
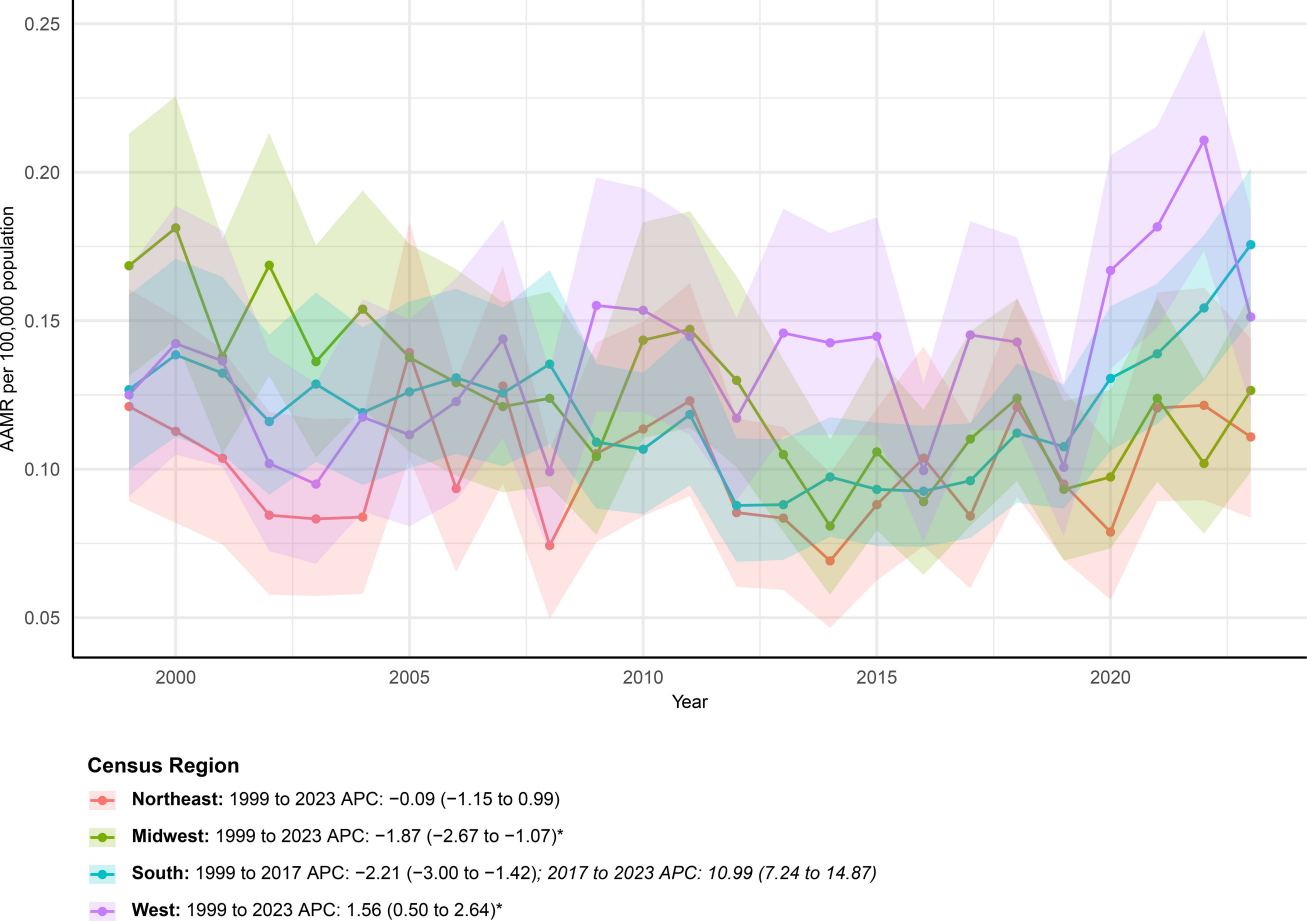
AAMRs of mortality involving hernia and diabetes among U.S. patients stratified by census region from 1999–2023.

Marked regional variation in mortality trends was observed across U.S. census regions (**Figure 3**). Between 1999 and 2023, AAMRs showed heterogeneous regional patterns across the four US census regions. In the Northeast, mortality remained relatively stable over the study period, with the age-adjusted rate decreasing slightly from 0.12 (95% CI 0.09–0.16) per 100,000 population in 1999 to 0.11 (95% CI 0.08–0.14) in 2023. The overall trend was not statistically significant, with an average annual percent change of −0.09% (95% CI −1.15 to 0.99).

The Midwest experienced a consistent and statistically significant decline in mortality. The age-adjusted rate decreased from 0.17 (95% CI 0.13–0.21) per 100,000 in 1999 to 0.13 (95% CI 0.10–0.16) in 2023, corresponding to an average annual percent change of −1.87% (95% CI −2.67 to −1.07). Joinpoint analysis indicated a sustained downward trajectory throughout the entire study period.

In contrast, the South demonstrated a more dynamic temporal pattern. Although mortality declined significantly from 1999 to 2017, this favorable trend reversed after 2017, with a marked increase observed through 2023. Over the full study period, the age-adjusted mortality rate rose from 0.13 (95% CI 0.10–0.16) to 0.18 (95% CI 0.15–0.20) per 100,000, yielding an overall average annual percent change of 0.93% (95% CI −0.06 to 1.94).

The West exhibited a gradual but statistically significant upward trend over time. The age-adjusted mortality rate increased from 0.13 (95% CI 0.09–0.17) per 100,000 population in 1999 to 0.15 (95% CI 0.12–0.19) in 2023, with an average annual percent change of 1.56% (95% CI 0.50 to 2.64), indicating a modest but persistent rise across the study period.

### 3.5 Race-stratified analyses

**Figure 4:**
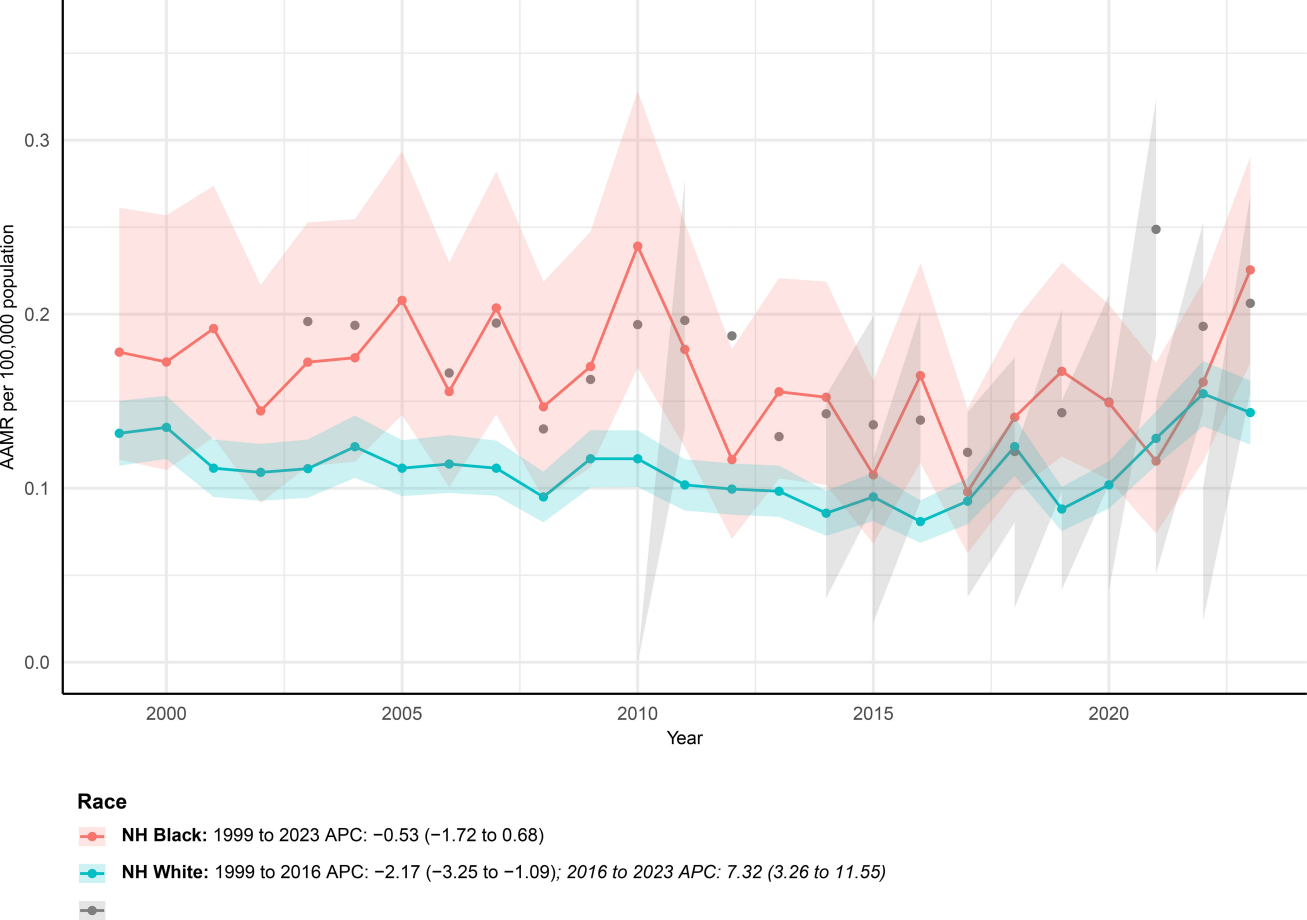
AAMRs of mortality involving hernia and diabetes among U.S. patients stratified by race from 1999–2023. Age-specific trend lines are not shown for individuals with races of Hispanic and NH other because of missing or suppressed data in CDC WONDER.

Substantial racial and ethnic disparities in mortality patterns were evident over the study period (**Figure 4**). Among Hispanic individuals, mortality increased markedly over the study period, with deaths rising from 13 in 1999 to 59 in 2023. The AAMR reached 0.21 per 100,000 population in 2023, indicating a clear upward pattern overall, although detailed Joinpoint segmentation could not be reliably estimated because of sparse early data.

Non-Hispanic Black individuals experienced relatively moderate temporal changes. Mortality increased from 26 deaths in 1999 to 67 deaths in 2023, with the age-adjusted mortality rate rising from 0.18 to 0.23 per 100,000 population. Trend analysis suggested a generally stable long-term pattern from 1999 to 2023, with an overall average annual percent change of −0.53 percent and no statistically significant inflection points identified.

In the Non-Hispanic White population, mortality increased from 205 deaths in 1999 to 284 deaths in 2023. The age-adjusted mortality rate rose slightly from 0.13 to 0.14 per 100,000 population. Joinpoint regression revealed a biphasic pattern, characterized by a significant decline between 1999 and 2016, followed by a pronounced upward shift from 2016 through 2023, indicating a reversal of earlier favorable trends.

For the Non-Hispanic Other group, mortality data were limited because of small case numbers, particularly in the early study years. Although the age-adjusted mortality rate in 2023 was low, temporal patterns could not be robustly characterized.

### 3.6 Urbanization-stratified analyses

**Figure 5:**
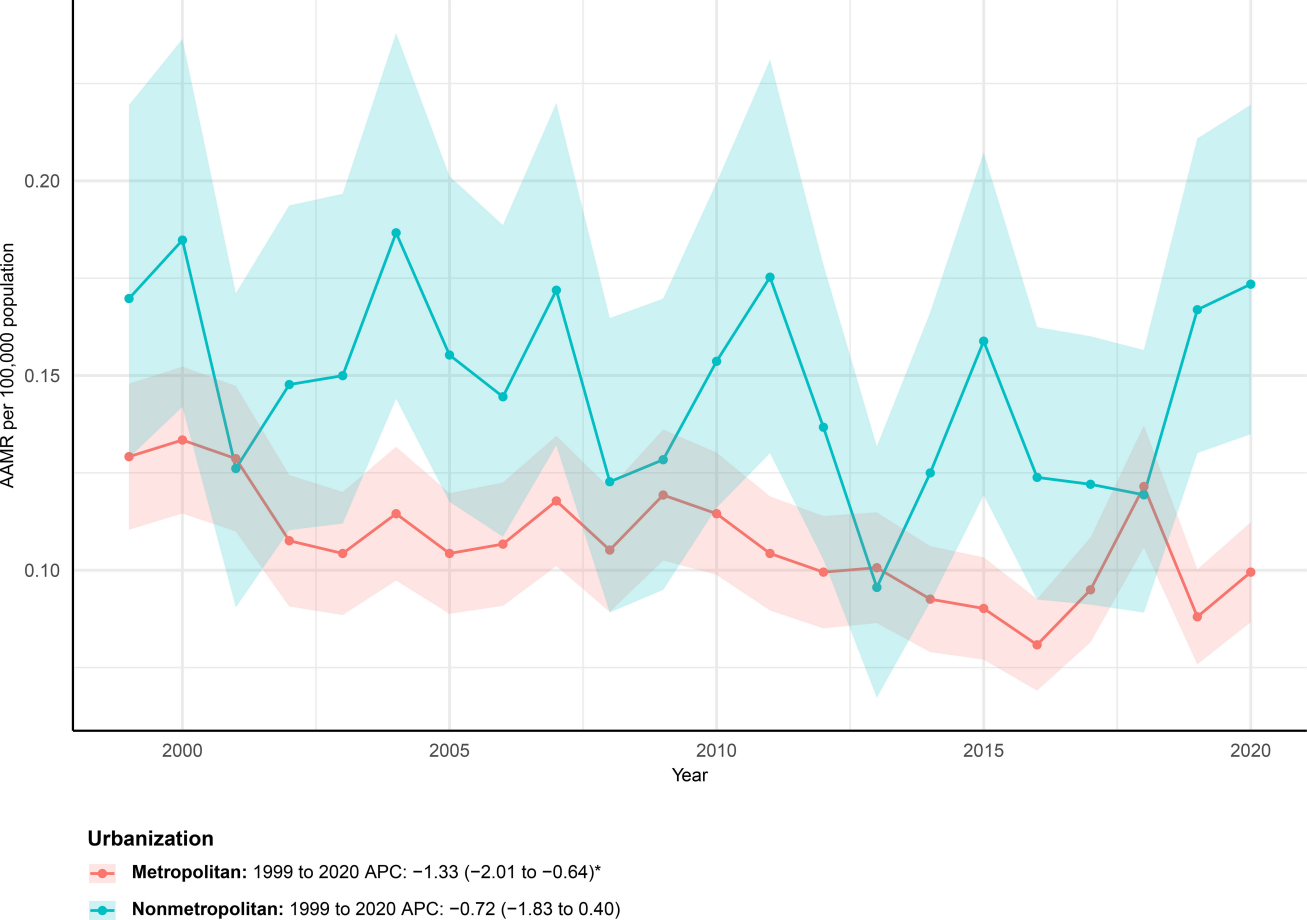
AAMRs of mortality involving hernia and diabetes among U.S. patients stratified by urbanization from 1999–2023.

Urbanization-stratified analyses revealed consistently lower and more pronounced mortality declines in metropolitan areas compared with nonmetropolitan areas. Significant differences in mortality trends were observed between metropolitan and nonmetropolitan areas during the study period, as illustrated in **Figure 5**. In metropolitan areas, the age-adjusted mortality rate declined steadily over time, decreasing from 0.13 (95% CI 0.11–0.15) per 100,000 population in 1999 to 0.10 (95% CI 0.09–0.11) in 2023. Joinpoint analysis demonstrated a significant long-term downward trend from 1999 through 2020, with an average annual percent change of −1.33% (95% CI −2.01 to −0.64), indicating sustained mortality reductions in metropolitan settings.

In contrast, nonmetropolitan areas exhibited a more attenuated decline in mortality. The age-adjusted mortality rate remained unchanged at 0.17 per 100,000 population between 1999 and 2023. Trend analysis suggested a modest overall decrease from 1999 to 2020, with an average annual percent change of −0.72% (95% CI −1.83 to 0.40), which did not reach statistical significance, reflecting comparatively slower improvements in nonmetropolitan areas.

## 4 Discussion

This study revealed substantial demographic and temporal heterogeneity in mortality involving hernia and diabetes from 1999 to 2023. Mortality patterns differed by sex, age, race, region, and urbanization, with recent upward reversals observed across several subgroups after 2017–2019. These findings underscore persistent population-level disparities and highlight the dynamic nature of mortality trends over time.

Mechanistically, the linkage between diabetes and worse hernia-related outcomes is biologically and clinically plausible. Diabetes impairs multiple reparative processes—reduced collagen deposition, impaired angiogenesis, microvascular dysfunction, and immune dysregulation—that collectively increase susceptibility to surgical site infection and wound breakdown after hernia repair, and that may worsen outcomes when strangulation or bowel ischemia occur (12). Evidence from systematic reviews and cohort studies confirms that diabetic patients are at elevated risk for surgical site infections and wound complications following hernia repair, with perioperative hyperglycemia itself being linked to a greater frequency of short-term adverse events in the ambulatory surgery setting (5, 8). Beyond wound-healing pathways, diabetes commonly coexists with other conditions—cardiovascular disease, chronic kidney disease, and obesity—that amplify perioperative risk and reduce physiologic reserve in the setting of emergency presentations such as incarcerated or strangulated hernias (13, 14); thus, diabetes is implicated in the elevated mortality observed in death-certificate co-occurrence studies through both direct pathways, such as increased tissue vulnerability and infection, and indirect pathways, reflected by a greater overall comorbidity burden (7).

The notable increase in incidence rates after 2019 represents a central finding of this study and warrants careful contextual interpretation. Although the ecological design precludes causal inference, this period coincides with substantial disruptions to healthcare delivery during the COVID-19 pandemic, including widespread delays in elective surgical care, postponed hernia repair, and reduced access to routine diabetes management. For individuals with concurrent hernia and diabetes, deferred surgical intervention and delayed clinical presentation may have increased the risk of complications in the setting of suboptimal glycemic control, a pattern reported across multiple surgical disciplines during the same period. The subsequent attenuation of mortality rates after 2021 may reflect partial normalization of healthcare access, resumption of elective procedures, and targeted efforts to address surgical backlogs. However, the persistence of elevated mortality in certain demographic subgroups suggests that recovery was uneven and that structural vulnerabilities related to metabolic health and healthcare access remain. Future studies integrating procedure-level surgical data, medication use, and healthcare utilization metrics will be critical for disentangling the relative contributions of biological risk, care delays, and health system disruption.

From a clinical and public-health perspective, our findings support several interrelated priorities. First, optimization of glycemic control before elective hernia repair deserves emphasis because both preoperative hyperglycemia and chronic poor glycemic control are associated with higher postoperative morbidity (15); same-day hyperglycemia in ambulatory hernia cohorts has been linked to increased short-term adverse events, reinforcing the role of perioperative glucose assessment and management (8). Second, for diabetic patients with risk stratification markers—such as advanced glycemic dysregulation, multiple comorbidities, or limited care access—a paradigm shift from watchful waiting to proactive elective repair may be warranted. This strategic approach could mitigate the transition to emergency presentations, which are associated with substantially higher morbidity and mortality (16). Third, enhanced perioperative risk stratification—integrating diabetes severity, renal and cardiovascular comorbidity, nutritional status and surgical urgency—should inform operative planning and postoperative surveillance (17). Finally, the marked geographic and racial/ethnic heterogeneity underscores the need to address structural barriers to surgical and chronic disease care, particularly in nonmetropolitan and socioeconomically disadvantaged communities.

While our study identifies key mortality patterns, its design has inherent limitations. Cause of death inaccuracies, unclear causal role of hernia, and absent clinical variables consequently limit definitive conclusions. Future work must therefore integrate operative and glycemic data from EHRs to clarify underlying mechanisms. A critical next step is to determine if targeted preoperative interventions directly mitigate risk in diabetic hernia patients.

## 5 Conclusion

In conclusion, this nationwide analysis from 1999 to 2023 indicates that mortality involving both hernia and diabetes in the United States exhibited substantial temporal heterogeneity, with no significant overall long-term change. Although mortality declined across several subgroups prior to 2017–2019, a pronounced reversal with rapidly increasing rates was observed in recent years. Persistent disparities across sex, age, race, region, and urbanization levels highlight unequal mortality burdens within this population. These findings emphasize the need for closer surveillance of recent adverse trends and for targeted strategies addressing high-risk groups in the management of patients with concurrent hernia and diabetes.

## Data Availability

The raw data supporting the conclusions of this article can be found here: https://wonder.cdc.gov/.
