# Supplementary material for: Long-term mortality trends in patients with concurrent hernia and diabetes in the United States
Source: Front Endocrinol (Lausanne). 2026 Feb 19;17:1723074. doi: 10.3389/fendo.2026.1723074 (PMC12960180; doi:10.3389/fendo.2026.1723074)
Supplement: Supplementary file 1 [file Table1.docx]

Supplementary table 1. Age-Adjusted Mortality Rates and Standard Errors by Characteristics Across Years.

| Characteristics | Year | AAMR | SE |
| --- | --- | --- | --- |
| Census Region_Midwest | 1999 | 0.168511738 | 0.020162368 |
| Census Region_Midwest | 2000 | 0.181240402 | 0.020213938 |
| Census Region_Midwest | 2001 | 0.137876348 | 0.017809862 |
| Census Region_Midwest | 2002 | 0.168678635 | 0.020232113 |
| Census Region_Midwest | 2003 | 0.136215408 | 0.017544486 |
| Census Region_Midwest | 2004 | 0.153882559 | 0.018135416 |
| Census Region_Midwest | 2005 | 0.137615795 | 0.01723571 |
| Census Region_Midwest | 2006 | 0.129162559 | 0.01706487 |
| Census Region_Midwest | 2007 | 0.121088477 | 0.015820199 |
| Census Region_Midwest | 2008 | 0.123844919 | 0.016152142 |
| Census Region_Midwest | 2009 | 0.104300606 | 0.014399271 |
| Census Region_Midwest | 2010 | 0.143437529 | 0.017989572 |
| Census Region_Midwest | 2011 | 0.147097198 | 0.017969652 |
| Census Region_Midwest | 2012 | 0.129899887 | 0.015928226 |
| Census Region_Midwest | 2013 | 0.104919334 | 0.014311048 |
| Census Region_Midwest | 2014 | 0.08083904 | 0.012736122 |
| Census Region_Midwest | 2015 | 0.105819592 | 0.014374001 |
| Census Region_Midwest | 2016 | 0.089028754 | 0.013528075 |
| Census Region_Midwest | 2017 | 0.110143318 | 0.016016847 |
| Census Region_Midwest | 2018 | 0.123893216 | 0.015137638 |
| Census Region_Midwest | 2019 | 0.09320656 | 0.013204253 |
| Census Region_Midwest | 2020 | 0.097366357 | 0.013134767 |
| Census Region_Midwest | 2021 | 0.123844919 | 0.015222398 |
| Census Region_Midwest | 2022 | 0.101902339 | 0.012837539 |
| Census Region_Midwest | 2023 | 0.126503739 | 0.014783579 |
| Census Region_Northeast | 1999 | 0.121088477 | 0.017397712 |
| Census Region_Northeast | 2000 | 0.112753841 | 0.01701968 |
| Census Region_Northeast | 2001 | 0.103681878 | 0.015912665 |
| Census Region_Northeast | 2002 | 0.084492772 | 0.014960047 |
| Census Region_Northeast | 2003 | 0.083237307 | 0.014413001 |
| Census Region_Northeast | 2004 | 0.083874044 | 0.014388349 |
| Census Region_Northeast | 2005 | 0.1392807 | 0.019524648 |
| Census Region_Northeast | 2006 | 0.093467113 | 0.015556288 |
| Census Region_Northeast | 2007 | 0.128004716 | 0.01799092 |
| Census Region_Northeast | 2008 | 0.074280975 | 0.013890961 |
| Census Region_Northeast | 2009 | 0.105179887 | 0.016423307 |
| Census Region_Northeast | 2010 | 0.113514523 | 0.015995846 |
| Census Region_Northeast | 2011 | 0.123013935 | 0.017648904 |
| Census Region_Northeast | 2012 | 0.085375021 | 0.01393299 |
| Census Region_Northeast | 2013 | 0.08349786 | 0.013325584 |
| Census Region_Northeast | 2014 | 0.069108256 | 0.012639517 |
| Census Region_Northeast | 2015 | 0.088033841 | 0.014064633 |
| Census Region_Northeast | 2016 | 0.103712166 | 0.016416568 |
| Census Region_Northeast | 2017 | 0.084235187 | 0.013480849 |
| Census Region_Northeast | 2018 | 0.120876221 | 0.016450377 |
| Census Region_Northeast | 2019 | 0.09496809 | 0.013953053 |
| Census Region_Northeast | 2020 | 0.078816957 | 0.012688645 |
| Census Region_Northeast | 2021 | 0.120615668 | 0.017172335 |
| Census Region_Northeast | 2022 | 0.121494949 | 0.017518684 |
| Census Region_Northeast | 2023 | 0.11085867 | 0.014827711 |
| Census Region_South | 1999 | 0.126764292 | 0.014573767 |
| Census Region_South | 2000 | 0.138495076 | 0.014883303 |
| Census Region_South | 2001 | 0.132328442 | 0.014822638 |
| Census Region_South | 2002 | 0.116031389 | 0.013350345 |
| Census Region_South | 2003 | 0.128641453 | 0.014085219 |
| Census Region_South | 2004 | 0.119048384 | 0.013182177 |
| Census Region_South | 2005 | 0.12603093 | 0.013910715 |
| Census Region_South | 2006 | 0.130779168 | 0.013792505 |
| Census Region_South | 2007 | 0.125606449 | 0.013254656 |
| Census Region_South | 2008 | 0.135363446 | 0.014549818 |
| Census Region_South | 2009 | 0.109097141 | 0.01208204 |
| Census Region_South | 2010 | 0.106698873 | 0.011818198 |
| Census Region_South | 2011 | 0.118411647 | 0.012811425 |
| Census Region_South | 2012 | 0.087773288 | 0.010270852 |
| Census Region_South | 2013 | 0.088033841 | 0.01004787 |
| Census Region_South | 2014 | 0.097366357 | 0.010267083 |
| Census Region_South | 2015 | 0.093188551 | 0.010309879 |
| Census Region_South | 2016 | 0.092569823 | 0.010093201 |
| Census Region_South | 2017 | 0.096107924 | 0.0098575 |
| Census Region_South | 2018 | 0.112162433 | 0.011937454 |
| Census Region_South | 2019 | 0.107578154 | 0.010637514 |
| Census Region_South | 2020 | 0.130566912 | 0.012411289 |
| Census Region_South | 2021 | 0.13885325 | 0.012021963 |
| Census Region_South | 2022 | 0.154289031 | 0.012480994 |
| Census Region_South | 2023 | 0.175594874 | 0.013035091 |
| Census Region_West | 1999 | 0.125002763 | 0.018904116 |
| Census Region_West | 2000 | 0.142297695 | 0.020506855 |
| Census Region_West | 2001 | 0.136454983 | 0.019435092 |
| Census Region_West | 2002 | 0.101884329 | 0.016372462 |
| Census Region_West | 2003 | 0.09496809 | 0.014900782 |
| Census Region_West | 2004 | 0.117529398 | 0.017560117 |
| Census Region_West | 2005 | 0.111593029 | 0.017065448 |
| Census Region_West | 2006 | 0.122753382 | 0.018272553 |
| Census Region_West | 2007 | 0.143816681 | 0.018120375 |
| Census Region_West | 2008 | 0.099127887 | 0.014947498 |
| Census Region_West | 2009 | 0.155120015 | 0.019329392 |
| Census Region_West | 2010 | 0.153507372 | 0.018575121 |
| Census Region_West | 2011 | 0.144695962 | 0.017922054 |
| Census Region_West | 2012 | 0.117171223 | 0.015176726 |
| Census Region_West | 2013 | 0.145835796 | 0.018893512 |
| Census Region_West | 2014 | 0.142558248 | 0.016807391 |
| Census Region_West | 2015 | 0.144695962 | 0.018041114 |
| Census Region_West | 2016 | 0.099504071 | 0.013086745 |
| Census Region_West | 2017 | 0.145199059 | 0.017406329 |
| Census Region_West | 2018 | 0.142800791 | 0.016086143 |
| Census Region_West | 2019 | 0.100643906 | 0.012676841 |
| Census Region_West | 2020 | 0.166902064 | 0.017815558 |
| Census Region_West | 2021 | 0.181598576 | 0.017305462 |
| Census Region_West | 2022 | 0.210787224 | 0.018957168 |
| Census Region_West | 2023 | 0.151254027 | 0.016375643 |
| Race_Hispanic | 1999 | NA | 0.047162454 |
| Race_Hispanic | 2000 | NA | 0.054943651 |
| Race_Hispanic | 2001 | NA | 0.043384441 |
| Race_Hispanic | 2002 | NA | 0.040542162 |
| Race_Hispanic | 2003 | 0.195799279 | 0.044398904 |
| Race_Hispanic | 2004 | 0.193592881 | 0.04447625 |
| Race_Hispanic | 2005 | NA | 0.034894746 |
| Race_Hispanic | 2006 | 0.166214061 | 0.037569814 |
| Race_Hispanic | 2007 | 0.194881602 | 0.039146699 |
| Race_Hispanic | 2008 | 0.134056716 | 0.032253508 |
| Race_Hispanic | 2009 | 0.162484681 | 0.0361265 |
| Race_Hispanic | 2010 | 0.193999353 | 0.036796386 |
| Race_Hispanic | 2011 | 0.19639762 | 0.0349319 |
| Race_Hispanic | 2012 | 0.187537913 | 0.034773648 |
| Race_Hispanic | 2013 | 0.129639334 | 0.02923266 |
| Race_Hispanic | 2014 | 0.142770504 | 0.028553583 |
| Race_Hispanic | 2015 | 0.136454983 | 0.026358331 |
| Race_Hispanic | 2016 | 0.139116771 | 0.026476431 |
| Race_Hispanic | 2017 | 0.120597658 | 0.022033663 |
| Race_Hispanic | 2018 | 0.121070467 | 0.022704348 |
| Race_Hispanic | 2019 | 0.143389232 | 0.025378442 |
| Race_Hispanic | 2020 | 0.149492497 | 0.026404714 |
| Race_Hispanic | 2021 | 0.248733047 | 0.033259011 |
| Race_Hispanic | 2022 | 0.193001473 | 0.026449306 |
| Race_Hispanic | 2023 | 0.206251242 | 0.027917822 |
| Race_NH Black | 1999 | 0.178187387 | 0.035250024 |
| Race_NH Black | 2000 | 0.172526614 | 0.035352609 |
| Race_NH Black | 2001 | 0.191727998 | 0.034914414 |
| Race_NH Black | 2002 | 0.1444174 | 0.029882965 |
| Race_NH Black | 2003 | 0.172428992 | 0.034028172 |
| Race_NH Black | 2004 | 0.174958136 | 0.033460238 |
| Race_NH Black | 2005 | 0.207918487 | 0.036867236 |
| Race_NH Black | 2006 | 0.155529455 | 0.031250883 |
| Race_NH Black | 2007 | 0.203574412 | 0.033985591 |
| Race_NH Black | 2008 | 0.146782411 | 0.030257702 |
| Race_NH Black | 2009 | 0.169937068 | 0.0326549 |
| Race_NH Black | 2010 | 0.239066671 | 0.039029666 |
| Race_NH Black | 2011 | 0.179754671 | 0.031161402 |
| Race_NH Black | 2012 | 0.116407574 | 0.026108389 |
| Race_NH Black | 2013 | 0.155410856 | 0.028030603 |
| Race_NH Black | 2014 | 0.152251907 | 0.028321209 |
| Race_NH Black | 2015 | 0.107678744 | 0.02247641 |
| Race_NH Black | 2016 | 0.164698042 | 0.027747528 |
| Race_NH Black | 2017 | 0.097887463 | 0.020021099 |
| Race_NH Black | 2018 | 0.140778709 | 0.023852023 |
| Race_NH Black | 2019 | 0.167159649 | 0.027263476 |
| Race_NH Black | 2020 | 0.149068015 | 0.024352503 |
| Race_NH Black | 2021 | 0.11557893 | 0.023654435 |
| Race_NH Black | 2022 | 0.160962727 | 0.025249283 |
| Race_NH Black | 2023 | 0.225419371 | 0.029179205 |
| Race_NH Other | 1999 | NA | NA |
| Race_NH Other | 2000 | NA | NA |
| Race_NH Other | 2001 | NA | NA |
| Race_NH Other | 2002 | NA | NA |
| Race_NH Other | 2003 | NA | NA |
| Race_NH Other | 2004 | NA | NA |
| Race_NH Other | 2005 | NA | NA |
| Race_NH Other | 2006 | NA | NA |
| Race_NH Other | 2007 | NA | NA |
| Race_NH Other | 2008 | NA | NA |
| Race_NH Other | 2009 | NA | NA |
| Race_NH Other | 2010 | NA | 0 |
| Race_NH Other | 2011 | NA | NA |
| Race_NH Other | 2012 | NA | NA |
| Race_NH Other | 2013 | NA | NA |
| Race_NH Other | 2014 | NA | 0.027094826 |
| Race_NH Other | 2015 | NA | 0.021604849 |
| Race_NH Other | 2016 | NA | NA |
| Race_NH Other | 2017 | NA | 0.024719041 |
| Race_NH Other | 2018 | NA | 0.020744657 |
| Race_NH Other | 2019 | NA | 0.024735911 |
| Race_NH Other | 2020 | NA | 0.025060545 |
| Race_NH Other | 2021 | NA | 0.023824918 |
| Race_NH Other | 2022 | NA | 0.017877998 |
| Race_NH Other | 2023 | NA | 0.018554538 |
| Race_NH White | 1999 | 0.131560827 | 0.009487123 |
| Race_NH White | 2000 | 0.134956974 | 0.009241001 |
| Race_NH White | 2001 | 0.111495408 | 0.008406979 |
| Race_NH White | 2002 | 0.109097141 | 0.008312136 |
| Race_NH White | 2003 | 0.111234855 | 0.008522617 |
| Race_NH White | 2004 | 0.123844919 | 0.009113561 |
| Race_NH White | 2005 | 0.111495408 | 0.008159596 |
| Race_NH White | 2006 | 0.113893675 | 0.008453325 |
| Race_NH White | 2007 | 0.111495408 | 0.008088498 |
| Race_NH White | 2008 | 0.09496809 | 0.007397107 |
| Race_NH White | 2009 | 0.11691067 | 0.008364508 |
| Race_NH White | 2010 | 0.11691067 | 0.008308809 |
| Race_NH White | 2011 | 0.101902339 | 0.007488171 |
| Race_NH White | 2012 | 0.099504071 | 0.007511178 |
| Race_NH White | 2013 | 0.098245638 | 0.007478737 |
| Race_NH White | 2014 | 0.085635574 | 0.006566247 |
| Race_NH White | 2015 | 0.09496809 | 0.007086642 |
| Race_NH White | 2016 | 0.08083904 | 0.006193384 |
| Race_NH White | 2017 | 0.092569823 | 0.006734152 |
| Race_NH White | 2018 | 0.123893216 | 0.008562897 |
| Race_NH White | 2019 | 0.088033841 | 0.006465362 |
| Race_NH White | 2020 | 0.101902339 | 0.006888817 |
| Race_NH White | 2021 | 0.128641453 | 0.007905156 |
| Race_NH White | 2022 | 0.154289031 | 0.009526956 |
| Race_NH White | 2023 | 0.143440497 | 0.009349155 |
| Sex_Both | 1999 | 0.136096808 | 0.008773642 |
| Sex_Both | 2000 | 0.145168771 | 0.00909138 |
| Sex_Both | 2001 | 0.124105472 | 0.008332059 |
| Sex_Both | 2002 | 0.11691067 | 0.008036517 |
| Sex_Both | 2003 | 0.116031389 | 0.007844565 |
| Sex_Both | 2004 | 0.126243186 | 0.008222712 |
| Sex_Both | 2005 | 0.119308937 | 0.007894162 |
| Sex_Both | 2006 | 0.111495408 | 0.007359864 |
| Sex_Both | 2007 | 0.119308937 | 0.007636151 |
| Sex_Both | 2008 | 0.101902339 | 0.007007839 |
| Sex_Both | 2009 | 0.11691067 | 0.007498426 |
| Sex_Both | 2010 | 0.137761714 | 0.008448728 |
| Sex_Both | 2011 | 0.125151649 | 0.007808559 |
| Sex_Both | 2012 | 0.101902339 | 0.006619003 |
| Sex_Both | 2013 | 0.098245638 | 0.006468995 |
| Sex_Both | 2014 | 0.09496809 | 0.006315536 |
| Sex_Both | 2015 | 0.10278162 | 0.006585492 |
| Sex_Both | 2016 | 0.090171556 | 0.005969112 |
| Sex_Both | 2017 | 0.09496809 | 0.006067726 |
| Sex_Both | 2018 | 0.121494949 | 0.00720699 |
| Sex_Both | 2019 | 0.097366357 | 0.006025288 |
| Sex_Both | 2020 | 0.104300606 | 0.006076973 |
| Sex_Both | 2021 | 0.128641453 | 0.006839525 |
| Sex_Both | 2022 | 0.151890764 | 0.007782647 |
| Sex_Both | 2023 | 0.156050561 | 0.00793433 |
| Sex_Female | 1999 | 0.160437656 | 0.012555545 |
| Sex_Female | 2000 | 0.145168771 | 0.011247569 |
| Sex_Female | 2001 | 0.159328109 | 0.013380314 |
| Sex_Female | 2002 | 0.126243186 | 0.010605744 |
| Sex_Female | 2003 | 0.121446652 | 0.010609189 |
| Sex_Female | 2004 | 0.131039721 | 0.010431606 |
| Sex_Female | 2005 | 0.11691067 | 0.009722412 |
| Sex_Female | 2006 | 0.121707205 | 0.010535094 |
| Sex_Female | 2007 | 0.158188275 | 0.012448614 |
| Sex_Female | 2008 | 0.117529398 | 0.010117722 |
| Sex_Female | 2009 | 0.119308937 | 0.009965746 |
| Sex_Female | 2010 | 0.134056716 | 0.010890918 |
| Sex_Female | 2011 | 0.120355115 | 0.010479122 |
| Sex_Female | 2012 | 0.112996385 | 0.009540582 |
| Sex_Female | 2013 | 0.100383352 | 0.009035004 |
| Sex_Female | 2014 | 0.097105804 | 0.008621102 |
| Sex_Female | 2015 | 0.101902339 | 0.008487538 |
| Sex_Female | 2016 | 0.08083904 | 0.007321967 |
| Sex_Female | 2017 | 0.092569823 | 0.00803921 |
| Sex_Female | 2018 | 0.112162433 | 0.008910819 |
| Sex_Female | 2019 | 0.097366357 | 0.00793624 |
| Sex_Female | 2020 | 0.099504071 | 0.008237457 |
| Sex_Female | 2021 | 0.13885325 | 0.009525277 |
| Sex_Female | 2022 | 0.128641453 | 0.008790956 |
| Sex_Female | 2023 | 0.151890764 | 0.010855215 |
| Sex_Male | 1999 | 0.114772956 | 0.013578192 |
| Sex_Male | 2000 | 0.130420993 | 0.014191022 |
| Sex_Male | 2001 | 0.117550376 | 0.013236537 |
| Sex_Male | 2002 | 0.097366357 | 0.011806295 |
| Sex_Male | 2003 | 0.121088477 | 0.013330473 |
| Sex_Male | 2004 | 0.097105804 | 0.011149839 |
| Sex_Male | 2005 | 0.132346451 | 0.013537108 |
| Sex_Male | 2006 | 0.120827924 | 0.012760294 |
| Sex_Male | 2007 | 0.121707205 | 0.012520535 |
| Sex_Male | 2008 | 0.091951095 | 0.010761348 |
| Sex_Male | 2009 | 0.11691067 | 0.011879221 |
| Sex_Male | 2010 | 0.137761714 | 0.013114408 |
| Sex_Male | 2011 | 0.141678967 | 0.012959043 |
| Sex_Male | 2012 | 0.104300606 | 0.010499233 |
| Sex_Male | 2013 | 0.100025178 | 0.010111746 |
| Sex_Male | 2014 | 0.126291483 | 0.012169445 |
| Sex_Male | 2015 | 0.11190188 | 0.011273336 |
| Sex_Male | 2016 | 0.105179887 | 0.009933051 |
| Sex_Male | 2017 | 0.118217401 | 0.011228286 |
| Sex_Male | 2018 | 0.126291483 | 0.011299876 |
| Sex_Male | 2019 | 0.100643906 | 0.009395169 |
| Sex_Male | 2020 | 0.126243186 | 0.010432598 |
| Sex_Male | 2021 | 0.135575702 | 0.011287768 |
| Sex_Male | 2022 | 0.158448828 | 0.011779236 |
| Sex_Male | 2023 | 0.151254027 | 0.011365751 |
| State_Alabama | 1999 | NA | NA |
| State_Alabama | 2000 | NA | NA |
| State_Alabama | 2001 | NA | NA |
| State_Alabama | 2002 | NA | NA |
| State_Alabama | 2003 | NA | NA |
| State_Alabama | 2004 | NA | NA |
| State_Alabama | 2005 | NA | NA |
| State_Alabama | 2006 | NA | NA |
| State_Alabama | 2007 | NA | NA |
| State_Alabama | 2008 | NA | NA |
| State_Alabama | 2009 | NA | NA |
| State_Alabama | 2010 | NA | NA |
| State_Alabama | 2011 | NA | NA |
| State_Alabama | 2012 | NA | NA |
| State_Alabama | 2013 | NA | NA |
| State_Alabama | 2014 | NA | NA |
| State_Alabama | 2015 | NA | NA |
| State_Alabama | 2016 | NA | NA |
| State_Alabama | 2017 | NA | NA |
| State_Alabama | 2018 | NA | NA |
| State_Alabama | 2019 | NA | NA |
| State_Alabama | 2020 | NA | NA |
| State_Alabama | 2021 | NA | NA |
| State_Alabama | 2022 | NA | NA |
| State_Alabama | 2023 | NA | NA |
| State_Alaska | 1999 | NA | NA |
| State_Alaska | 2000 | NA | 0 |
| State_Alaska | 2001 | NA | 0 |
| State_Alaska | 2002 | NA | NA |
| State_Alaska | 2003 | NA | 0 |
| State_Alaska | 2004 | NA | NA |
| State_Alaska | 2005 | NA | 0 |
| State_Alaska | 2006 | NA | 0 |
| State_Alaska | 2007 | NA | 0 |
| State_Alaska | 2008 | NA | NA |
| State_Alaska | 2009 | NA | 0 |
| State_Alaska | 2010 | NA | 0 |
| State_Alaska | 2011 | NA | 0 |
| State_Alaska | 2012 | NA | NA |
| State_Alaska | 2013 | NA | NA |
| State_Alaska | 2014 | NA | 0 |
| State_Alaska | 2015 | NA | 0 |
| State_Alaska | 2016 | NA | 0 |
| State_Alaska | 2017 | NA | 0 |
| State_Alaska | 2018 | NA | 0 |
| State_Alaska | 2019 | NA | NA |
| State_Alaska | 2020 | NA | NA |
| State_Alaska | 2021 | NA | NA |
| State_Alaska | 2022 | NA | 0 |
| State_Alaska | 2023 | NA | 0 |
| State_Arizona | 1999 | NA | NA |
| State_Arizona | 2000 | NA | NA |
| State_Arizona | 2001 | NA | 0 |
| State_Arizona | 2002 | NA | NA |
| State_Arizona | 2003 | NA | NA |
| State_Arizona | 2004 | NA | NA |
| State_Arizona | 2005 | NA | NA |
| State_Arizona | 2006 | NA | NA |
| State_Arizona | 2007 | NA | NA |
| State_Arizona | 2008 | NA | NA |
| State_Arizona | 2009 | NA | NA |
| State_Arizona | 2010 | NA | NA |
| State_Arizona | 2011 | NA | NA |
| State_Arizona | 2012 | NA | NA |
| State_Arizona | 2013 | NA | NA |
| State_Arizona | 2014 | NA | 0.061057091 |
| State_Arizona | 2015 | NA | NA |
| State_Arizona | 2016 | NA | NA |
| State_Arizona | 2017 | NA | 0.053702029 |
| State_Arizona | 2018 | NA | NA |
| State_Arizona | 2019 | NA | NA |
| State_Arizona | 2020 | NA | NA |
| State_Arizona | 2021 | NA | NA |
| State_Arizona | 2022 | NA | NA |
| State_Arizona | 2023 | NA | NA |
| State_Arkansas | 1999 | NA | 0 |
| State_Arkansas | 2000 | NA | NA |
| State_Arkansas | 2001 | NA | NA |
| State_Arkansas | 2002 | NA | NA |
| State_Arkansas | 2003 | NA | NA |
| State_Arkansas | 2004 | NA | NA |
| State_Arkansas | 2005 | NA | NA |
| State_Arkansas | 2006 | NA | 0 |
| State_Arkansas | 2007 | NA | NA |
| State_Arkansas | 2008 | NA | 0 |
| State_Arkansas | 2009 | NA | NA |
| State_Arkansas | 2010 | NA | NA |
| State_Arkansas | 2011 | NA | NA |
| State_Arkansas | 2012 | NA | NA |
| State_Arkansas | 2013 | NA | NA |
| State_Arkansas | 2014 | NA | NA |
| State_Arkansas | 2015 | NA | NA |
| State_Arkansas | 2016 | NA | NA |
| State_Arkansas | 2017 | NA | NA |
| State_Arkansas | 2018 | NA | NA |
| State_Arkansas | 2019 | NA | NA |
| State_Arkansas | 2020 | NA | NA |
| State_Arkansas | 2021 | NA | NA |
| State_Arkansas | 2022 | NA | NA |
| State_Arkansas | 2023 | NA | NA |
| State_California | 1999 | 0.160437656 | 0.029877577 |
| State_California | 2000 | 0.177684291 | 0.031528939 |
| State_California | 2001 | 0.20876748 | 0.03469293 |
| State_California | 2002 | 0.144872199 | 0.027915283 |
| State_California | 2003 | 0.123468734 | 0.024728862 |
| State_California | 2004 | 0.172035795 | 0.029302377 |
| State_California | 2005 | 0.131137342 | 0.025839046 |
| State_California | 2006 | 0.142297695 | 0.026340471 |
| State_California | 2007 | 0.193805107 | 0.030698765 |
| State_California | 2008 | 0.146651738 | 0.02530614 |
| State_California | 2009 | 0.188699722 | 0.0296757 |
| State_California | 2010 | 0.198390413 | 0.029735755 |
| State_California | 2011 | 0.174715593 | 0.026947414 |
| State_California | 2012 | 0.150207849 | 0.025375163 |
| State_California | 2013 | 0.127122467 | 0.022020236 |
| State_California | 2014 | 0.162460736 | 0.025677662 |
| State_California | 2015 | 0.137779723 | 0.024585679 |
| State_California | 2016 | 0.142491941 | 0.023189375 |
| State_California | 2017 | 0.128053013 | 0.021102284 |
| State_California | 2018 | 0.15149657 | 0.023844092 |
| State_California | 2019 | 0.124366025 | 0.021174622 |
| State_California | 2020 | 0.206872938 | 0.027598937 |
| State_California | 2021 | 0.221945635 | 0.02834123 |
| State_California | 2022 | 0.187701842 | 0.024998976 |
| State_California | 2023 | 0.163603538 | 0.023684902 |
| State_Colorado | 1999 | NA | NA |
| State_Colorado | 2000 | NA | NA |
| State_Colorado | 2001 | NA | NA |
| State_Colorado | 2002 | NA | NA |
| State_Colorado | 2003 | NA | NA |
| State_Colorado | 2004 | NA | NA |
| State_Colorado | 2005 | NA | NA |
| State_Colorado | 2006 | NA | NA |
| State_Colorado | 2007 | NA | NA |
| State_Colorado | 2008 | NA | NA |
| State_Colorado | 2009 | NA | NA |
| State_Colorado | 2010 | NA | NA |
| State_Colorado | 2011 | NA | NA |
| State_Colorado | 2012 | NA | NA |
| State_Colorado | 2013 | NA | NA |
| State_Colorado | 2014 | NA | NA |
| State_Colorado | 2015 | NA | NA |
| State_Colorado | 2016 | NA | NA |
| State_Colorado | 2017 | NA | NA |
| State_Colorado | 2018 | NA | 0.082587767 |
| State_Colorado | 2019 | NA | NA |
| State_Colorado | 2020 | NA | NA |
| State_Colorado | 2021 | NA | NA |
| State_Colorado | 2022 | NA | 0.087069994 |
| State_Colorado | 2023 | NA | NA |
| State_Connecticut | 1999 | NA | NA |
| State_Connecticut | 2000 | NA | NA |
| State_Connecticut | 2001 | NA | NA |
| State_Connecticut | 2002 | NA | NA |
| State_Connecticut | 2003 | NA | NA |
| State_Connecticut | 2004 | NA | NA |
| State_Connecticut | 2005 | NA | NA |
| State_Connecticut | 2006 | NA | NA |
| State_Connecticut | 2007 | NA | NA |
| State_Connecticut | 2008 | NA | NA |
| State_Connecticut | 2009 | NA | NA |
| State_Connecticut | 2010 | NA | NA |
| State_Connecticut | 2011 | NA | NA |
| State_Connecticut | 2012 | NA | NA |
| State_Connecticut | 2013 | NA | NA |
| State_Connecticut | 2014 | NA | 0 |
| State_Connecticut | 2015 | NA | NA |
| State_Connecticut | 2016 | NA | NA |
| State_Connecticut | 2017 | NA | NA |
| State_Connecticut | 2018 | NA | NA |
| State_Connecticut | 2019 | NA | NA |
| State_Connecticut | 2020 | NA | NA |
| State_Connecticut | 2021 | NA | NA |
| State_Connecticut | 2022 | NA | NA |
| State_Connecticut | 2023 | NA | NA |
| State_Delaware | 1999 | NA | 0 |
| State_Delaware | 2000 | NA | 0 |
| State_Delaware | 2001 | NA | NA |
| State_Delaware | 2002 | NA | 0 |
| State_Delaware | 2003 | NA | 0 |
| State_Delaware | 2004 | NA | NA |
| State_Delaware | 2005 | NA | 0 |
| State_Delaware | 2006 | NA | 0 |
| State_Delaware | 2007 | NA | NA |
| State_Delaware | 2008 | NA | 0 |
| State_Delaware | 2009 | NA | NA |
| State_Delaware | 2010 | NA | NA |
| State_Delaware | 2011 | NA | 0 |
| State_Delaware | 2012 | NA | NA |
| State_Delaware | 2013 | NA | NA |
| State_Delaware | 2014 | NA | NA |
| State_Delaware | 2015 | NA | NA |
| State_Delaware | 2016 | NA | 0 |
| State_Delaware | 2017 | NA | 0 |
| State_Delaware | 2018 | NA | NA |
| State_Delaware | 2019 | NA | 0 |
| State_Delaware | 2020 | NA | NA |
| State_Delaware | 2021 | NA | NA |
| State_Delaware | 2022 | NA | NA |
| State_Delaware | 2023 | NA | 0 |
| State_District of Columbia | 1999 | NA | 0 |
| State_District of Columbia | 2000 | NA | NA |
| State_District of Columbia | 2001 | NA | NA |
| State_District of Columbia | 2002 | NA | 0 |
| State_District of Columbia | 2003 | NA | 0 |
| State_District of Columbia | 2004 | NA | 0 |
| State_District of Columbia | 2005 | NA | 0 |
| State_District of Columbia | 2006 | NA | NA |
| State_District of Columbia | 2007 | NA | NA |
| State_District of Columbia | 2008 | NA | 0 |
| State_District of Columbia | 2009 | NA | 0 |
| State_District of Columbia | 2010 | NA | NA |
| State_District of Columbia | 2011 | NA | 0 |
| State_District of Columbia | 2012 | NA | NA |
| State_District of Columbia | 2013 | NA | NA |
| State_District of Columbia | 2014 | NA | 0 |
| State_District of Columbia | 2015 | NA | 0 |
| State_District of Columbia | 2016 | NA | 0 |
| State_District of Columbia | 2017 | NA | 0 |
| State_District of Columbia | 2018 | NA | NA |
| State_District of Columbia | 2019 | NA | 0 |
| State_District of Columbia | 2020 | NA | NA |
| State_District of Columbia | 2021 | NA | NA |
| State_District of Columbia | 2022 | NA | 0 |
| State_District of Columbia | 2023 | NA | NA |
| State_Florida | 1999 | NA | NA |
| State_Florida | 2000 | NA | NA |
| State_Florida | 2001 | NA | 0.023182131 |
| State_Florida | 2002 | NA | NA |
| State_Florida | 2003 | NA | NA |
| State_Florida | 2004 | NA | NA |
| State_Florida | 2005 | NA | NA |
| State_Florida | 2006 | NA | 0.023225266 |
| State_Florida | 2007 | NA | 0.025053451 |
| State_Florida | 2008 | NA | 0.020985905 |
| State_Florida | 2009 | NA | NA |
| State_Florida | 2010 | NA | 0.018612732 |
| State_Florida | 2011 | NA | 0.01814679 |
| State_Florida | 2012 | NA | NA |
| State_Florida | 2013 | NA | 0.01923632 |
| State_Florida | 2014 | NA | 0.024573883 |
| State_Florida | 2015 | NA | 0.018069239 |
| State_Florida | 2016 | NA | 0.017332637 |
| State_Florida | 2017 | NA | 0.019342733 |
| State_Florida | 2018 | NA | 0.017292105 |
| State_Florida | 2019 | NA | 0.022621499 |
| State_Florida | 2020 | NA | 0.014948077 |
| State_Florida | 2021 | 0.109903114 | 0.027012645 |
| State_Florida | 2022 | 0.128053013 | 0.026899838 |
| State_Florida | 2023 | 0.133225732 | 0.026774957 |
| State_Georgia | 1999 | NA | NA |
| State_Georgia | 2000 | NA | 0.079006533 |
| State_Georgia | 2001 | NA | 0.070387888 |
| State_Georgia | 2002 | NA | NA |
| State_Georgia | 2003 | NA | NA |
| State_Georgia | 2004 | NA | 0.077646478 |
| State_Georgia | 2005 | NA | 0.072455319 |
| State_Georgia | 2006 | NA | 0.069451063 |
| State_Georgia | 2007 | NA | 0.065702679 |
| State_Georgia | 2008 | NA | NA |
| State_Georgia | 2009 | NA | NA |
| State_Georgia | 2010 | NA | NA |
| State_Georgia | 2011 | NA | NA |
| State_Georgia | 2012 | NA | NA |
| State_Georgia | 2013 | NA | NA |
| State_Georgia | 2014 | NA | NA |
| State_Georgia | 2015 | NA | NA |
| State_Georgia | 2016 | NA | NA |
| State_Georgia | 2017 | NA | NA |
| State_Georgia | 2018 | NA | NA |
| State_Georgia | 2019 | NA | NA |
| State_Georgia | 2020 | NA | NA |
| State_Georgia | 2021 | NA | NA |
| State_Georgia | 2022 | NA | 0.042967734 |
| State_Georgia | 2023 | NA | NA |
| State_Hawaii | 1999 | NA | NA |
| State_Hawaii | 2000 | NA | 0 |
| State_Hawaii | 2001 | NA | NA |
| State_Hawaii | 2002 | NA | NA |
| State_Hawaii | 2003 | NA | NA |
| State_Hawaii | 2004 | NA | NA |
| State_Hawaii | 2005 | NA | 0 |
| State_Hawaii | 2006 | NA | NA |
| State_Hawaii | 2007 | NA | NA |
| State_Hawaii | 2008 | NA | 0 |
| State_Hawaii | 2009 | NA | NA |
| State_Hawaii | 2010 | NA | 0 |
| State_Hawaii | 2011 | NA | 0 |
| State_Hawaii | 2012 | NA | NA |
| State_Hawaii | 2013 | NA | NA |
| State_Hawaii | 2014 | NA | NA |
| State_Hawaii | 2015 | NA | 0 |
| State_Hawaii | 2016 | NA | 0 |
| State_Hawaii | 2017 | NA | 0 |
| State_Hawaii | 2018 | NA | NA |
| State_Hawaii | 2019 | NA | NA |
| State_Hawaii | 2020 | NA | NA |
| State_Hawaii | 2021 | NA | 0 |
| State_Hawaii | 2022 | NA | NA |
| State_Hawaii | 2023 | NA | NA |
| State_Idaho | 1999 | NA | NA |
| State_Idaho | 2000 | NA | 0 |
| State_Idaho | 2001 | NA | 0 |
| State_Idaho | 2002 | NA | NA |
| State_Idaho | 2003 | NA | 0 |
| State_Idaho | 2004 | NA | NA |
| State_Idaho | 2005 | NA | 0 |
| State_Idaho | 2006 | NA | NA |
| State_Idaho | 2007 | NA | NA |
| State_Idaho | 2008 | NA | 0 |
| State_Idaho | 2009 | NA | NA |
| State_Idaho | 2010 | NA | NA |
| State_Idaho | 2011 | NA | NA |
| State_Idaho | 2012 | NA | NA |
| State_Idaho | 2013 | NA | NA |
| State_Idaho | 2014 | NA | NA |
| State_Idaho | 2015 | NA | NA |
| State_Idaho | 2016 | NA | NA |
| State_Idaho | 2017 | NA | NA |
| State_Idaho | 2018 | NA | NA |
| State_Idaho | 2019 | NA | 0 |
| State_Idaho | 2020 | NA | NA |
| State_Idaho | 2021 | NA | NA |
| State_Idaho | 2022 | NA | NA |
| State_Idaho | 2023 | NA | NA |
| State_Illinois | 1999 | NA | 0.041470807 |
| State_Illinois | 2000 | NA | NA |
| State_Illinois | 2001 | NA | NA |
| State_Illinois | 2002 | NA | 0.047768329 |
| State_Illinois | 2003 | NA | 0.044782053 |
| State_Illinois | 2004 | NA | 0.055476654 |
| State_Illinois | 2005 | NA | NA |
| State_Illinois | 2006 | NA | 0.045701924 |
| State_Illinois | 2007 | NA | NA |
| State_Illinois | 2008 | NA | 0.046725906 |
| State_Illinois | 2009 | NA | NA |
| State_Illinois | 2010 | NA | 0.038125701 |
| State_Illinois | 2011 | NA | NA |
| State_Illinois | 2012 | NA | NA |
| State_Illinois | 2013 | NA | 0.046598074 |
| State_Illinois | 2014 | NA | NA |
| State_Illinois | 2015 | NA | NA |
| State_Illinois | 2016 | NA | NA |
| State_Illinois | 2017 | NA | NA |
| State_Illinois | 2018 | NA | 0.034234399 |
| State_Illinois | 2019 | NA | NA |
| State_Illinois | 2020 | NA | NA |
| State_Illinois | 2021 | NA | 0.031744485 |
| State_Illinois | 2022 | NA | 0.042690671 |
| State_Illinois | 2023 | NA | NA |
| State_Indiana | 1999 | NA | NA |
| State_Indiana | 2000 | NA | NA |
| State_Indiana | 2001 | NA | NA |
| State_Indiana | 2002 | NA | 0.092044774 |
| State_Indiana | 2003 | NA | NA |
| State_Indiana | 2004 | NA | 0.078193635 |
| State_Indiana | 2005 | NA | NA |
| State_Indiana | 2006 | NA | NA |
| State_Indiana | 2007 | NA | NA |
| State_Indiana | 2008 | NA | NA |
| State_Indiana | 2009 | NA | NA |
| State_Indiana | 2010 | NA | NA |
| State_Indiana | 2011 | NA | NA |
| State_Indiana | 2012 | NA | 0.085260582 |
| State_Indiana | 2013 | NA | NA |
| State_Indiana | 2014 | NA | NA |
| State_Indiana | 2015 | NA | NA |
| State_Indiana | 2016 | NA | NA |
| State_Indiana | 2017 | NA | NA |
| State_Indiana | 2018 | NA | NA |
| State_Indiana | 2019 | NA | NA |
| State_Indiana | 2020 | NA | NA |
| State_Indiana | 2021 | NA | 0.055849482 |
| State_Indiana | 2022 | NA | NA |
| State_Indiana | 2023 | NA | 0.06553293 |
| State_Iowa | 1999 | NA | NA |
| State_Iowa | 2000 | NA | NA |
| State_Iowa | 2001 | NA | NA |
| State_Iowa | 2002 | NA | NA |
| State_Iowa | 2003 | NA | NA |
| State_Iowa | 2004 | NA | NA |
| State_Iowa | 2005 | NA | NA |
| State_Iowa | 2006 | NA | NA |
| State_Iowa | 2007 | NA | NA |
| State_Iowa | 2008 | NA | NA |
| State_Iowa | 2009 | NA | NA |
| State_Iowa | 2010 | NA | NA |
| State_Iowa | 2011 | NA | NA |
| State_Iowa | 2012 | NA | NA |
| State_Iowa | 2013 | NA | NA |
| State_Iowa | 2014 | NA | NA |
| State_Iowa | 2015 | NA | NA |
| State_Iowa | 2016 | NA | NA |
| State_Iowa | 2017 | NA | NA |
| State_Iowa | 2018 | NA | NA |
| State_Iowa | 2019 | NA | NA |
| State_Iowa | 2020 | NA | NA |
| State_Iowa | 2021 | NA | NA |
| State_Iowa | 2022 | NA | NA |
| State_Iowa | 2023 | NA | NA |
| State_Kansas | 1999 | NA | NA |
| State_Kansas | 2000 | NA | NA |
| State_Kansas | 2001 | NA | NA |
| State_Kansas | 2002 | NA | NA |
| State_Kansas | 2003 | NA | NA |
| State_Kansas | 2004 | NA | NA |
| State_Kansas | 2005 | NA | NA |
| State_Kansas | 2006 | NA | NA |
| State_Kansas | 2007 | NA | 0 |
| State_Kansas | 2008 | NA | NA |
| State_Kansas | 2009 | NA | NA |
| State_Kansas | 2010 | NA | NA |
| State_Kansas | 2011 | NA | NA |
| State_Kansas | 2012 | NA | NA |
| State_Kansas | 2013 | NA | 0 |
| State_Kansas | 2014 | NA | NA |
| State_Kansas | 2015 | NA | NA |
| State_Kansas | 2016 | NA | NA |
| State_Kansas | 2017 | NA | 0 |
| State_Kansas | 2018 | NA | NA |
| State_Kansas | 2019 | NA | 0 |
| State_Kansas | 2020 | NA | NA |
| State_Kansas | 2021 | NA | NA |
| State_Kansas | 2022 | NA | 0 |
| State_Kansas | 2023 | NA | NA |
| State_Kentucky | 1999 | NA | NA |
| State_Kentucky | 2000 | NA | NA |
| State_Kentucky | 2001 | NA | NA |
| State_Kentucky | 2002 | NA | NA |
| State_Kentucky | 2003 | NA | NA |
| State_Kentucky | 2004 | NA | NA |
| State_Kentucky | 2005 | NA | NA |
| State_Kentucky | 2006 | NA | NA |
| State_Kentucky | 2007 | NA | NA |
| State_Kentucky | 2008 | NA | NA |
| State_Kentucky | 2009 | NA | NA |
| State_Kentucky | 2010 | NA | NA |
| State_Kentucky | 2011 | NA | NA |
| State_Kentucky | 2012 | NA | NA |
| State_Kentucky | 2013 | NA | NA |
| State_Kentucky | 2014 | NA | NA |
| State_Kentucky | 2015 | NA | NA |
| State_Kentucky | 2016 | NA | NA |
| State_Kentucky | 2017 | NA | NA |
| State_Kentucky | 2018 | NA | NA |
| State_Kentucky | 2019 | NA | 0.102270328 |
| State_Kentucky | 2020 | NA | NA |
| State_Kentucky | 2021 | NA | NA |
| State_Kentucky | 2022 | NA | NA |
| State_Kentucky | 2023 | NA | 0.097612046 |
| State_Louisiana | 1999 | NA | NA |
| State_Louisiana | 2000 | NA | NA |
| State_Louisiana | 2001 | NA | NA |
| State_Louisiana | 2002 | NA | NA |
| State_Louisiana | 2003 | NA | NA |
| State_Louisiana | 2004 | NA | NA |
| State_Louisiana | 2005 | NA | NA |
| State_Louisiana | 2006 | NA | NA |
| State_Louisiana | 2007 | NA | 0 |
| State_Louisiana | 2008 | NA | NA |
| State_Louisiana | 2009 | NA | NA |
| State_Louisiana | 2010 | NA | NA |
| State_Louisiana | 2011 | NA | NA |
| State_Louisiana | 2012 | NA | 0 |
| State_Louisiana | 2013 | NA | NA |
| State_Louisiana | 2014 | NA | NA |
| State_Louisiana | 2015 | NA | NA |
| State_Louisiana | 2016 | NA | NA |
| State_Louisiana | 2017 | NA | NA |
| State_Louisiana | 2018 | NA | NA |
| State_Louisiana | 2019 | NA | NA |
| State_Louisiana | 2020 | NA | NA |
| State_Louisiana | 2021 | NA | NA |
| State_Louisiana | 2022 | NA | NA |
| State_Louisiana | 2023 | NA | NA |
| State_Maine | 1999 | NA | NA |
| State_Maine | 2000 | NA | 0 |
| State_Maine | 2001 | NA | NA |
| State_Maine | 2002 | NA | NA |
| State_Maine | 2003 | NA | NA |
| State_Maine | 2004 | NA | 0 |
| State_Maine | 2005 | NA | NA |
| State_Maine | 2006 | NA | NA |
| State_Maine | 2007 | NA | NA |
| State_Maine | 2008 | NA | NA |
| State_Maine | 2009 | NA | NA |
| State_Maine | 2010 | NA | NA |
| State_Maine | 2011 | NA | 0 |
| State_Maine | 2012 | NA | NA |
| State_Maine | 2013 | NA | NA |
| State_Maine | 2014 | NA | NA |
| State_Maine | 2015 | NA | NA |
| State_Maine | 2016 | NA | NA |
| State_Maine | 2017 | NA | NA |
| State_Maine | 2018 | NA | NA |
| State_Maine | 2019 | NA | NA |
| State_Maine | 2020 | NA | 0 |
| State_Maine | 2021 | NA | NA |
| State_Maine | 2022 | NA | NA |
| State_Maine | 2023 | NA | NA |
| State_Maryland | 1999 | NA | 0.105886735 |
| State_Maryland | 2000 | NA | NA |
| State_Maryland | 2001 | NA | NA |
| State_Maryland | 2002 | NA | NA |
| State_Maryland | 2003 | NA | NA |
| State_Maryland | 2004 | NA | NA |
| State_Maryland | 2005 | NA | NA |
| State_Maryland | 2006 | NA | NA |
| State_Maryland | 2007 | NA | NA |
| State_Maryland | 2008 | NA | NA |
| State_Maryland | 2009 | NA | NA |
| State_Maryland | 2010 | NA | NA |
| State_Maryland | 2011 | NA | NA |
| State_Maryland | 2012 | NA | NA |
| State_Maryland | 2013 | NA | NA |
| State_Maryland | 2014 | NA | NA |
| State_Maryland | 2015 | NA | NA |
| State_Maryland | 2016 | NA | NA |
| State_Maryland | 2017 | NA | NA |
| State_Maryland | 2018 | NA | NA |
| State_Maryland | 2019 | NA | NA |
| State_Maryland | 2020 | NA | NA |
| State_Maryland | 2021 | NA | 0.064789018 |
| State_Maryland | 2022 | NA | NA |
| State_Maryland | 2023 | NA | 0.079277752 |
| State_Massachusetts | 1999 | NA | NA |
| State_Massachusetts | 2000 | NA | NA |
| State_Massachusetts | 2001 | NA | NA |
| State_Massachusetts | 2002 | NA | NA |
| State_Massachusetts | 2003 | NA | NA |
| State_Massachusetts | 2004 | NA | NA |
| State_Massachusetts | 2005 | NA | NA |
| State_Massachusetts | 2006 | NA | NA |
| State_Massachusetts | 2007 | NA | NA |
| State_Massachusetts | 2008 | NA | NA |
| State_Massachusetts | 2009 | NA | NA |
| State_Massachusetts | 2010 | NA | NA |
| State_Massachusetts | 2011 | NA | NA |
| State_Massachusetts | 2012 | NA | NA |
| State_Massachusetts | 2013 | NA | NA |
| State_Massachusetts | 2014 | NA | NA |
| State_Massachusetts | 2015 | NA | NA |
| State_Massachusetts | 2016 | NA | NA |
| State_Massachusetts | 2017 | NA | NA |
| State_Massachusetts | 2018 | NA | NA |
| State_Massachusetts | 2019 | NA | NA |
| State_Massachusetts | 2020 | NA | NA |
| State_Massachusetts | 2021 | NA | NA |
| State_Massachusetts | 2022 | NA | NA |
| State_Massachusetts | 2023 | NA | NA |
| State_Michigan | 1999 | NA | 0.0501189 |
| State_Michigan | 2000 | NA | NA |
| State_Michigan | 2001 | NA | 0.057246693 |
| State_Michigan | 2002 | NA | NA |
| State_Michigan | 2003 | NA | NA |
| State_Michigan | 2004 | NA | 0.046996176 |
| State_Michigan | 2005 | NA | NA |
| State_Michigan | 2006 | NA | 0.047393936 |
| State_Michigan | 2007 | NA | NA |
| State_Michigan | 2008 | NA | NA |
| State_Michigan | 2009 | NA | NA |
| State_Michigan | 2010 | NA | NA |
| State_Michigan | 2011 | NA | 0.04290783 |
| State_Michigan | 2012 | NA | NA |
| State_Michigan | 2013 | NA | NA |
| State_Michigan | 2014 | NA | NA |
| State_Michigan | 2015 | NA | NA |
| State_Michigan | 2016 | NA | NA |
| State_Michigan | 2017 | NA | NA |
| State_Michigan | 2018 | NA | NA |
| State_Michigan | 2019 | NA | NA |
| State_Michigan | 2020 | NA | NA |
| State_Michigan | 2021 | NA | 0.037718131 |
| State_Michigan | 2022 | NA | 0.038180896 |
| State_Michigan | 2023 | NA | NA |
| State_Minnesota | 1999 | NA | NA |
| State_Minnesota | 2000 | NA | 0.098459519 |
| State_Minnesota | 2001 | NA | NA |
| State_Minnesota | 2002 | NA | NA |
| State_Minnesota | 2003 | NA | NA |
| State_Minnesota | 2004 | NA | 0.091439429 |
| State_Minnesota | 2005 | NA | NA |
| State_Minnesota | 2006 | NA | NA |
| State_Minnesota | 2007 | NA | NA |
| State_Minnesota | 2008 | NA | NA |
| State_Minnesota | 2009 | NA | NA |
| State_Minnesota | 2010 | NA | NA |
| State_Minnesota | 2011 | NA | NA |
| State_Minnesota | 2012 | NA | 0.093782609 |
| State_Minnesota | 2013 | NA | NA |
| State_Minnesota | 2014 | NA | NA |
| State_Minnesota | 2015 | NA | NA |
| State_Minnesota | 2016 | NA | NA |
| State_Minnesota | 2017 | NA | NA |
| State_Minnesota | 2018 | NA | 0.072403567 |
| State_Minnesota | 2019 | NA | NA |
| State_Minnesota | 2020 | NA | 0.07913825 |
| State_Minnesota | 2021 | NA | NA |
| State_Minnesota | 2022 | NA | 0.062084896 |
| State_Minnesota | 2023 | NA | 0.082079055 |
| State_Mississippi | 1999 | NA | NA |
| State_Mississippi | 2000 | NA | NA |
| State_Mississippi | 2001 | NA | NA |
| State_Mississippi | 2002 | NA | NA |
| State_Mississippi | 2003 | NA | NA |
| State_Mississippi | 2004 | NA | NA |
| State_Mississippi | 2005 | NA | NA |
| State_Mississippi | 2006 | NA | NA |
| State_Mississippi | 2007 | NA | NA |
| State_Mississippi | 2008 | NA | NA |
| State_Mississippi | 2009 | NA | NA |
| State_Mississippi | 2010 | NA | NA |
| State_Mississippi | 2011 | NA | NA |
| State_Mississippi | 2012 | NA | NA |
| State_Mississippi | 2013 | NA | NA |
| State_Mississippi | 2014 | NA | NA |
| State_Mississippi | 2015 | NA | NA |
| State_Mississippi | 2016 | NA | 0 |
| State_Mississippi | 2017 | NA | NA |
| State_Mississippi | 2018 | NA | 0 |
| State_Mississippi | 2019 | NA | NA |
| State_Mississippi | 2020 | NA | NA |
| State_Mississippi | 2021 | NA | NA |
| State_Mississippi | 2022 | NA | NA |
| State_Mississippi | 2023 | NA | NA |
| State_Missouri | 1999 | NA | NA |
| State_Missouri | 2000 | NA | NA |
| State_Missouri | 2001 | NA | NA |
| State_Missouri | 2002 | NA | NA |
| State_Missouri | 2003 | NA | NA |
| State_Missouri | 2004 | NA | NA |
| State_Missouri | 2005 | NA | NA |
| State_Missouri | 2006 | NA | NA |
| State_Missouri | 2007 | NA | NA |
| State_Missouri | 2008 | NA | 0.083137691 |
| State_Missouri | 2009 | NA | NA |
| State_Missouri | 2010 | NA | NA |
| State_Missouri | 2011 | NA | NA |
| State_Missouri | 2012 | NA | NA |
| State_Missouri | 2013 | NA | NA |
| State_Missouri | 2014 | NA | NA |
| State_Missouri | 2015 | NA | NA |
| State_Missouri | 2016 | NA | NA |
| State_Missouri | 2017 | NA | NA |
| State_Missouri | 2018 | NA | NA |
| State_Missouri | 2019 | NA | NA |
| State_Missouri | 2020 | NA | NA |
| State_Missouri | 2021 | NA | NA |
| State_Missouri | 2022 | NA | NA |
| State_Missouri | 2023 | NA | 0.060468496 |
| State_Montana | 1999 | NA | NA |
| State_Montana | 2000 | NA | NA |
| State_Montana | 2001 | NA | NA |
| State_Montana | 2002 | NA | 0 |
| State_Montana | 2003 | NA | NA |
| State_Montana | 2004 | NA | 0 |
| State_Montana | 2005 | NA | 0 |
| State_Montana | 2006 | NA | NA |
| State_Montana | 2007 | NA | NA |
| State_Montana | 2008 | NA | NA |
| State_Montana | 2009 | NA | NA |
| State_Montana | 2010 | NA | 0 |
| State_Montana | 2011 | NA | NA |
| State_Montana | 2012 | NA | NA |
| State_Montana | 2013 | NA | 0 |
| State_Montana | 2014 | NA | NA |
| State_Montana | 2015 | NA | 0 |
| State_Montana | 2016 | NA | NA |
| State_Montana | 2017 | NA | NA |
| State_Montana | 2018 | NA | NA |
| State_Montana | 2019 | NA | NA |
| State_Montana | 2020 | NA | NA |
| State_Montana | 2021 | NA | NA |
| State_Montana | 2022 | NA | NA |
| State_Montana | 2023 | NA | NA |
| State_Nebraska | 1999 | NA | NA |
| State_Nebraska | 2000 | NA | NA |
| State_Nebraska | 2001 | NA | 0 |
| State_Nebraska | 2002 | NA | 0 |
| State_Nebraska | 2003 | NA | NA |
| State_Nebraska | 2004 | NA | NA |
| State_Nebraska | 2005 | NA | NA |
| State_Nebraska | 2006 | NA | NA |
| State_Nebraska | 2007 | NA | NA |
| State_Nebraska | 2008 | NA | NA |
| State_Nebraska | 2009 | NA | NA |
| State_Nebraska | 2010 | NA | NA |
| State_Nebraska | 2011 | NA | NA |
| State_Nebraska | 2012 | NA | NA |
| State_Nebraska | 2013 | NA | 0 |
| State_Nebraska | 2014 | NA | NA |
| State_Nebraska | 2015 | NA | NA |
| State_Nebraska | 2016 | NA | NA |
| State_Nebraska | 2017 | NA | NA |
| State_Nebraska | 2018 | NA | NA |
| State_Nebraska | 2019 | NA | NA |
| State_Nebraska | 2020 | NA | 0 |
| State_Nebraska | 2021 | NA | NA |
| State_Nebraska | 2022 | NA | NA |
| State_Nebraska | 2023 | NA | 0 |
| State_Nevada | 1999 | NA | 0 |
| State_Nevada | 2000 | NA | NA |
| State_Nevada | 2001 | NA | NA |
| State_Nevada | 2002 | NA | 0 |
| State_Nevada | 2003 | NA | NA |
| State_Nevada | 2004 | NA | 0 |
| State_Nevada | 2005 | NA | 0 |
| State_Nevada | 2006 | NA | 0 |
| State_Nevada | 2007 | NA | 0 |
| State_Nevada | 2008 | NA | 0 |
| State_Nevada | 2009 | NA | NA |
| State_Nevada | 2010 | NA | 0 |
| State_Nevada | 2011 | NA | 0 |
| State_Nevada | 2012 | NA | NA |
| State_Nevada | 2013 | NA | 0 |
| State_Nevada | 2014 | NA | NA |
| State_Nevada | 2015 | NA | NA |
| State_Nevada | 2016 | NA | NA |
| State_Nevada | 2017 | NA | 0 |
| State_Nevada | 2018 | NA | NA |
| State_Nevada | 2019 | NA | NA |
| State_Nevada | 2020 | NA | 0 |
| State_Nevada | 2021 | NA | 0 |
| State_Nevada | 2022 | NA | NA |
| State_Nevada | 2023 | NA | 0 |
| State_New Hampshire | 1999 | NA | NA |
| State_New Hampshire | 2000 | NA | NA |
| State_New Hampshire | 2001 | NA | NA |
| State_New Hampshire | 2002 | NA | NA |
| State_New Hampshire | 2003 | NA | NA |
| State_New Hampshire | 2004 | NA | 0 |
| State_New Hampshire | 2005 | NA | NA |
| State_New Hampshire | 2006 | NA | NA |
| State_New Hampshire | 2007 | NA | 0 |
| State_New Hampshire | 2008 | NA | NA |
| State_New Hampshire | 2009 | NA | NA |
| State_New Hampshire | 2010 | NA | NA |
| State_New Hampshire | 2011 | NA | NA |
| State_New Hampshire | 2012 | NA | NA |
| State_New Hampshire | 2013 | NA | NA |
| State_New Hampshire | 2014 | NA | 0 |
| State_New Hampshire | 2015 | NA | NA |
| State_New Hampshire | 2016 | NA | NA |
| State_New Hampshire | 2017 | NA | NA |
| State_New Hampshire | 2018 | NA | NA |
| State_New Hampshire | 2019 | NA | NA |
| State_New Hampshire | 2020 | NA | NA |
| State_New Hampshire | 2021 | NA | NA |
| State_New Hampshire | 2022 | NA | 0 |
| State_New Hampshire | 2023 | NA | NA |
| State_New Jersey | 1999 | NA | NA |
| State_New Jersey | 2000 | NA | 0.055510468 |
| State_New Jersey | 2001 | NA | NA |
| State_New Jersey | 2002 | NA | NA |
| State_New Jersey | 2003 | NA | NA |
| State_New Jersey | 2004 | NA | NA |
| State_New Jersey | 2005 | NA | NA |
| State_New Jersey | 2006 | NA | NA |
| State_New Jersey | 2007 | NA | NA |
| State_New Jersey | 2008 | NA | NA |
| State_New Jersey | 2009 | NA | NA |
| State_New Jersey | 2010 | NA | NA |
| State_New Jersey | 2011 | NA | NA |
| State_New Jersey | 2012 | NA | NA |
| State_New Jersey | 2013 | NA | 0.051353364 |
| State_New Jersey | 2014 | NA | 0.051050649 |
| State_New Jersey | 2015 | NA | NA |
| State_New Jersey | 2016 | NA | NA |
| State_New Jersey | 2017 | NA | NA |
| State_New Jersey | 2018 | NA | NA |
| State_New Jersey | 2019 | NA | NA |
| State_New Jersey | 2020 | NA | NA |
| State_New Jersey | 2021 | NA | NA |
| State_New Jersey | 2022 | NA | NA |
| State_New Jersey | 2023 | NA | NA |
| State_New Mexico | 1999 | NA | 0 |
| State_New Mexico | 2000 | NA | NA |
| State_New Mexico | 2001 | NA | NA |
| State_New Mexico | 2002 | NA | NA |
| State_New Mexico | 2003 | NA | NA |
| State_New Mexico | 2004 | NA | 0 |
| State_New Mexico | 2005 | NA | NA |
| State_New Mexico | 2006 | NA | NA |
| State_New Mexico | 2007 | NA | NA |
| State_New Mexico | 2008 | NA | NA |
| State_New Mexico | 2009 | NA | NA |
| State_New Mexico | 2010 | NA | NA |
| State_New Mexico | 2011 | NA | NA |
| State_New Mexico | 2012 | NA | NA |
| State_New Mexico | 2013 | NA | NA |
| State_New Mexico | 2014 | NA | NA |
| State_New Mexico | 2015 | NA | NA |
| State_New Mexico | 2016 | NA | NA |
| State_New Mexico | 2017 | NA | NA |
| State_New Mexico | 2018 | NA | NA |
| State_New Mexico | 2019 | NA | NA |
| State_New Mexico | 2020 | NA | NA |
| State_New Mexico | 2021 | NA | NA |
| State_New Mexico | 2022 | NA | NA |
| State_New Mexico | 2023 | NA | NA |
| State_New York | 1999 | NA | 0.029308869 |
| State_New York | 2000 | NA | 0.026195047 |
| State_New York | 2001 | NA | 0.031302016 |
| State_New York | 2002 | NA | 0.02898858 |
| State_New York | 2003 | NA | NA |
| State_New York | 2004 | NA | 0.028106939 |
| State_New York | 2005 | NA | 0.023849306 |
| State_New York | 2006 | NA | 0.026883969 |
| State_New York | 2007 | 0.173815335 | 0.037424614 |
| State_New York | 2008 | NA | NA |
| State_New York | 2009 | NA | 0.024985456 |
| State_New York | 2010 | NA | 0.027734367 |
| State_New York | 2011 | NA | 0.028410642 |
| State_New York | 2012 | NA | 0.028002737 |
| State_New York | 2013 | NA | 0.019327294 |
| State_New York | 2014 | NA | 0.021637789 |
| State_New York | 2015 | NA | 0.029792497 |
| State_New York | 2016 | NA | 0.022547601 |
| State_New York | 2017 | NA | 0.026198292 |
| State_New York | 2018 | 0.169919058 | 0.034009954 |
| State_New York | 2019 | 0.120188218 | 0.026495428 |
| State_New York | 2020 | NA | 0.0207786 |
| State_New York | 2021 | NA | 0.028999871 |
| State_New York | 2022 | 0.162008276 | 0.034141034 |
| State_New York | 2023 | 0.110646415 | 0.02613221 |
| State_North Carolina | 1999 | NA | NA |
| State_North Carolina | 2000 | NA | 0.078275271 |
| State_North Carolina | 2001 | NA | NA |
| State_North Carolina | 2002 | NA | 0.079431861 |
| State_North Carolina | 2003 | NA | NA |
| State_North Carolina | 2004 | NA | NA |
| State_North Carolina | 2005 | NA | 0.061712793 |
| State_North Carolina | 2006 | NA | NA |
| State_North Carolina | 2007 | NA | NA |
| State_North Carolina | 2008 | NA | 0.058028418 |
| State_North Carolina | 2009 | NA | 0.05127006 |
| State_North Carolina | 2010 | NA | 0.061890361 |
| State_North Carolina | 2011 | NA | NA |
| State_North Carolina | 2012 | NA | 0.057256502 |
| State_North Carolina | 2013 | NA | NA |
| State_North Carolina | 2014 | NA | NA |
| State_North Carolina | 2015 | NA | NA |
| State_North Carolina | 2016 | NA | NA |
| State_North Carolina | 2017 | NA | 0.043539658 |
| State_North Carolina | 2018 | NA | NA |
| State_North Carolina | 2019 | NA | NA |
| State_North Carolina | 2020 | NA | NA |
| State_North Carolina | 2021 | NA | 0.038568287 |
| State_North Carolina | 2022 | NA | 0.046398285 |
| State_North Carolina | 2023 | NA | 0.040774146 |
| State_North Dakota | 1999 | NA | 0 |
| State_North Dakota | 2000 | NA | NA |
| State_North Dakota | 2001 | NA | NA |
| State_North Dakota | 2002 | NA | NA |
| State_North Dakota | 2003 | NA | 0 |
| State_North Dakota | 2004 | NA | 0 |
| State_North Dakota | 2005 | NA | 0 |
| State_North Dakota | 2006 | NA | NA |
| State_North Dakota | 2007 | NA | NA |
| State_North Dakota | 2008 | NA | NA |
| State_North Dakota | 2009 | NA | 0 |
| State_North Dakota | 2010 | NA | NA |
| State_North Dakota | 2011 | NA | NA |
| State_North Dakota | 2012 | NA | NA |
| State_North Dakota | 2013 | NA | 0 |
| State_North Dakota | 2014 | NA | NA |
| State_North Dakota | 2015 | NA | NA |
| State_North Dakota | 2016 | NA | NA |
| State_North Dakota | 2017 | NA | 0 |
| State_North Dakota | 2018 | NA | 0 |
| State_North Dakota | 2019 | NA | NA |
| State_North Dakota | 2020 | NA | 0 |
| State_North Dakota | 2021 | NA | NA |
| State_North Dakota | 2022 | NA | NA |
| State_North Dakota | 2023 | NA | NA |
| State_Ohio | 1999 | NA | 0.056293698 |
| State_Ohio | 2000 | 0.385089411 | 0.071520033 |
| State_Ohio | 2001 | 0.307221011 | 0.064554733 |
| State_Ohio | 2002 | NA | 0.053756193 |
| State_Ohio | 2003 | NA | 0.047622559 |
| State_Ohio | 2004 | NA | 0.044376178 |
| State_Ohio | 2005 | NA | 0.048085414 |
| State_Ohio | 2006 | NA | 0.05298528 |
| State_Ohio | 2007 | 0.236749618 | 0.053048929 |
| State_Ohio | 2008 | NA | 0.038977576 |
| State_Ohio | 2009 | NA | 0.047112367 |
| State_Ohio | 2010 | 0.254287295 | 0.056333465 |
| State_Ohio | 2011 | NA | 0.051081861 |
| State_Ohio | 2012 | NA | 0.041248893 |
| State_Ohio | 2013 | NA | 0.040220332 |
| State_Ohio | 2014 | NA | NA |
| State_Ohio | 2015 | NA | 0.039755771 |
| State_Ohio | 2016 | NA | NA |
| State_Ohio | 2017 | NA | 0.035313194 |
| State_Ohio | 2018 | NA | 0.031542771 |
| State_Ohio | 2019 | NA | NA |
| State_Ohio | 2020 | NA | NA |
| State_Ohio | 2021 | NA | NA |
| State_Ohio | 2022 | NA | 0.03191533 |
| State_Ohio | 2023 | NA | NA |
| State_Oklahoma | 1999 | NA | NA |
| State_Oklahoma | 2000 | NA | NA |
| State_Oklahoma | 2001 | NA | NA |
| State_Oklahoma | 2002 | NA | NA |
| State_Oklahoma | 2003 | NA | 0.133994847 |
| State_Oklahoma | 2004 | NA | NA |
| State_Oklahoma | 2005 | NA | NA |
| State_Oklahoma | 2006 | NA | NA |
| State_Oklahoma | 2007 | NA | NA |
| State_Oklahoma | 2008 | NA | NA |
| State_Oklahoma | 2009 | NA | NA |
| State_Oklahoma | 2010 | NA | NA |
| State_Oklahoma | 2011 | NA | NA |
| State_Oklahoma | 2012 | NA | NA |
| State_Oklahoma | 2013 | NA | NA |
| State_Oklahoma | 2014 | NA | 0.118769681 |
| State_Oklahoma | 2015 | NA | 0.110158058 |
| State_Oklahoma | 2016 | NA | 0.116644981 |
| State_Oklahoma | 2017 | NA | 0.120760443 |
| State_Oklahoma | 2018 | NA | 0.125382339 |
| State_Oklahoma | 2019 | NA | 0.105276586 |
| State_Oklahoma | 2020 | NA | 0.122281921 |
| State_Oklahoma | 2021 | NA | 0.136837763 |
| State_Oklahoma | 2022 | NA | 0.134670383 |
| State_Oklahoma | 2023 | 0.639293692 | 0.14562181 |
| State_Oregon | 1999 | NA | NA |
| State_Oregon | 2000 | NA | NA |
| State_Oregon | 2001 | NA | NA |
| State_Oregon | 2002 | NA | NA |
| State_Oregon | 2003 | NA | NA |
| State_Oregon | 2004 | NA | NA |
| State_Oregon | 2005 | NA | NA |
| State_Oregon | 2006 | NA | NA |
| State_Oregon | 2007 | NA | NA |
| State_Oregon | 2008 | NA | NA |
| State_Oregon | 2009 | NA | NA |
| State_Oregon | 2010 | NA | NA |
| State_Oregon | 2011 | NA | NA |
| State_Oregon | 2012 | NA | NA |
| State_Oregon | 2013 | NA | NA |
| State_Oregon | 2014 | NA | NA |
| State_Oregon | 2015 | NA | NA |
| State_Oregon | 2016 | NA | NA |
| State_Oregon | 2017 | NA | NA |
| State_Oregon | 2018 | NA | NA |
| State_Oregon | 2019 | NA | NA |
| State_Oregon | 2020 | NA | NA |
| State_Oregon | 2021 | NA | NA |
| State_Oregon | 2022 | NA | 0.121695627 |
| State_Oregon | 2023 | NA | NA |
| State_Pennsylvania | 1999 | 0.223042081 | 0.048974341 |
| State_Pennsylvania | 2000 | NA | 0.038640327 |
| State_Pennsylvania | 2001 | NA | 0.040059825 |
| State_Pennsylvania | 2002 | NA | NA |
| State_Pennsylvania | 2003 | NA | 0.034728922 |
| State_Pennsylvania | 2004 | NA | 0.038664074 |
| State_Pennsylvania | 2005 | 0.276347411 | 0.056812163 |
| State_Pennsylvania | 2006 | NA | 0.034950163 |
| State_Pennsylvania | 2007 | NA | 0.035989103 |
| State_Pennsylvania | 2008 | NA | 0.031237582 |
| State_Pennsylvania | 2009 | NA | NA |
| State_Pennsylvania | 2010 | NA | 0.032655539 |
| State_Pennsylvania | 2011 | NA | 0.038467234 |
| State_Pennsylvania | 2012 | NA | 0.032256268 |
| State_Pennsylvania | 2013 | NA | 0.041050189 |
| State_Pennsylvania | 2014 | NA | NA |
| State_Pennsylvania | 2015 | NA | 0.034243935 |
| State_Pennsylvania | 2016 | NA | NA |
| State_Pennsylvania | 2017 | NA | NA |
| State_Pennsylvania | 2018 | NA | 0.030670166 |
| State_Pennsylvania | 2019 | NA | NA |
| State_Pennsylvania | 2020 | NA | 0.02928085 |
| State_Pennsylvania | 2021 | NA | 0.039171297 |
| State_Pennsylvania | 2022 | NA | 0.044226222 |
| State_Pennsylvania | 2023 | 0.21322665 | 0.049774251 |
| State_Rhode Island | 1999 | NA | NA |
| State_Rhode Island | 2000 | NA | NA |
| State_Rhode Island | 2001 | NA | NA |
| State_Rhode Island | 2002 | NA | NA |
| State_Rhode Island | 2003 | NA | 0 |
| State_Rhode Island | 2004 | NA | NA |
| State_Rhode Island | 2005 | NA | NA |
| State_Rhode Island | 2006 | NA | NA |
| State_Rhode Island | 2007 | NA | 0 |
| State_Rhode Island | 2008 | NA | 0 |
| State_Rhode Island | 2009 | NA | NA |
| State_Rhode Island | 2010 | NA | NA |
| State_Rhode Island | 2011 | NA | 0 |
| State_Rhode Island | 2012 | NA | NA |
| State_Rhode Island | 2013 | NA | NA |
| State_Rhode Island | 2014 | NA | NA |
| State_Rhode Island | 2015 | NA | NA |
| State_Rhode Island | 2016 | NA | 0 |
| State_Rhode Island | 2017 | NA | 0 |
| State_Rhode Island | 2018 | NA | NA |
| State_Rhode Island | 2019 | NA | 0 |
| State_Rhode Island | 2020 | NA | NA |
| State_Rhode Island | 2021 | NA | NA |
| State_Rhode Island | 2022 | NA | NA |
| State_Rhode Island | 2023 | NA | NA |
| State_South Carolina | 1999 | NA | NA |
| State_South Carolina | 2000 | NA | NA |
| State_South Carolina | 2001 | NA | NA |
| State_South Carolina | 2002 | NA | NA |
| State_South Carolina | 2003 | NA | NA |
| State_South Carolina | 2004 | NA | NA |
| State_South Carolina | 2005 | NA | NA |
| State_South Carolina | 2006 | NA | NA |
| State_South Carolina | 2007 | NA | NA |
| State_South Carolina | 2008 | NA | NA |
| State_South Carolina | 2009 | NA | NA |
| State_South Carolina | 2010 | NA | NA |
| State_South Carolina | 2011 | NA | NA |
| State_South Carolina | 2012 | NA | NA |
| State_South Carolina | 2013 | NA | NA |
| State_South Carolina | 2014 | NA | NA |
| State_South Carolina | 2015 | NA | NA |
| State_South Carolina | 2016 | NA | NA |
| State_South Carolina | 2017 | NA | NA |
| State_South Carolina | 2018 | NA | NA |
| State_South Carolina | 2019 | NA | NA |
| State_South Carolina | 2020 | NA | NA |
| State_South Carolina | 2021 | NA | NA |
| State_South Carolina | 2022 | NA | 0.075771411 |
| State_South Carolina | 2023 | NA | NA |
| State_South Dakota | 1999 | NA | NA |
| State_South Dakota | 2000 | NA | NA |
| State_South Dakota | 2001 | NA | 0 |
| State_South Dakota | 2002 | NA | NA |
| State_South Dakota | 2003 | NA | NA |
| State_South Dakota | 2004 | NA | NA |
| State_South Dakota | 2005 | NA | NA |
| State_South Dakota | 2006 | NA | 0 |
| State_South Dakota | 2007 | NA | 0 |
| State_South Dakota | 2008 | NA | 0 |
| State_South Dakota | 2009 | NA | 0 |
| State_South Dakota | 2010 | NA | NA |
| State_South Dakota | 2011 | NA | NA |
| State_South Dakota | 2012 | NA | 0 |
| State_South Dakota | 2013 | NA | NA |
| State_South Dakota | 2014 | NA | NA |
| State_South Dakota | 2015 | NA | NA |
| State_South Dakota | 2016 | NA | 0 |
| State_South Dakota | 2017 | NA | NA |
| State_South Dakota | 2018 | NA | NA |
| State_South Dakota | 2019 | NA | NA |
| State_South Dakota | 2020 | NA | NA |
| State_South Dakota | 2021 | NA | NA |
| State_South Dakota | 2022 | NA | NA |
| State_South Dakota | 2023 | NA | NA |
| State_Tennessee | 1999 | NA | NA |
| State_Tennessee | 2000 | NA | NA |
| State_Tennessee | 2001 | NA | NA |
| State_Tennessee | 2002 | NA | NA |
| State_Tennessee | 2003 | NA | NA |
| State_Tennessee | 2004 | NA | NA |
| State_Tennessee | 2005 | NA | NA |
| State_Tennessee | 2006 | NA | NA |
| State_Tennessee | 2007 | NA | NA |
| State_Tennessee | 2008 | NA | NA |
| State_Tennessee | 2009 | NA | 0.080770814 |
| State_Tennessee | 2010 | NA | NA |
| State_Tennessee | 2011 | NA | NA |
| State_Tennessee | 2012 | NA | NA |
| State_Tennessee | 2013 | NA | NA |
| State_Tennessee | 2014 | NA | NA |
| State_Tennessee | 2015 | NA | NA |
| State_Tennessee | 2016 | NA | NA |
| State_Tennessee | 2017 | NA | NA |
| State_Tennessee | 2018 | NA | NA |
| State_Tennessee | 2019 | NA | NA |
| State_Tennessee | 2020 | NA | 0.058670637 |
| State_Tennessee | 2021 | NA | NA |
| State_Tennessee | 2022 | NA | NA |
| State_Tennessee | 2023 | NA | 0.06670463 |
| State_Texas | 1999 | NA | 0.042430096 |
| State_Texas | 2000 | NA | 0.033804926 |
| State_Texas | 2001 | NA | 0.034669412 |
| State_Texas | 2002 | NA | NA |
| State_Texas | 2003 | 0.175994413 | 0.04081613 |
| State_Texas | 2004 | 0.162675959 | 0.038479912 |
| State_Texas | 2005 | 0.161681047 | 0.037498421 |
| State_Texas | 2006 | 0.174194487 | 0.037824344 |
| State_Texas | 2007 | 0.206775316 | 0.042620984 |
| State_Texas | 2008 | NA | 0.029364542 |
| State_Texas | 2009 | 0.14428949 | 0.034203894 |
| State_Texas | 2010 | NA | 0.030911551 |
| State_Texas | 2011 | 0.175855427 | 0.035165736 |
| State_Texas | 2012 | NA | 0.026263789 |
| State_Texas | 2013 | NA | 0.023606584 |
| State_Texas | 2014 | NA | 0.028598469 |
| State_Texas | 2015 | 0.133680531 | 0.028187653 |
| State_Texas | 2016 | 0.127501619 | 0.027873869 |
| State_Texas | 2017 | 0.131970267 | 0.029585828 |
| State_Texas | 2018 | 0.148476607 | 0.029345494 |
| State_Texas | 2019 | 0.142509951 | 0.028814295 |
| State_Texas | 2020 | 0.117026301 | 0.025506605 |
| State_Texas | 2021 | 0.174615003 | 0.031790533 |
| State_Texas | 2022 | 0.207509675 | 0.033841735 |
| State_Texas | 2023 | 0.223657841 | 0.035798668 |
| State_Utah | 1999 | NA | NA |
| State_Utah | 2000 | NA | 0 |
| State_Utah | 2001 | NA | NA |
| State_Utah | 2002 | NA | NA |
| State_Utah | 2003 | NA | NA |
| State_Utah | 2004 | NA | 0 |
| State_Utah | 2005 | NA | 0 |
| State_Utah | 2006 | NA | NA |
| State_Utah | 2007 | NA | NA |
| State_Utah | 2008 | NA | NA |
| State_Utah | 2009 | NA | NA |
| State_Utah | 2010 | NA | NA |
| State_Utah | 2011 | NA | 0 |
| State_Utah | 2012 | NA | NA |
| State_Utah | 2013 | NA | NA |
| State_Utah | 2014 | NA | 0 |
| State_Utah | 2015 | NA | NA |
| State_Utah | 2016 | NA | 0 |
| State_Utah | 2017 | NA | NA |
| State_Utah | 2018 | NA | 0 |
| State_Utah | 2019 | NA | NA |
| State_Utah | 2020 | NA | NA |
| State_Utah | 2021 | NA | NA |
| State_Utah | 2022 | NA | NA |
| State_Utah | 2023 | NA | NA |
| State_Vermont | 1999 | NA | NA |
| State_Vermont | 2000 | NA | 0 |
| State_Vermont | 2001 | NA | NA |
| State_Vermont | 2002 | NA | 0 |
| State_Vermont | 2003 | NA | NA |
| State_Vermont | 2004 | NA | NA |
| State_Vermont | 2005 | NA | NA |
| State_Vermont | 2006 | NA | NA |
| State_Vermont | 2007 | NA | NA |
| State_Vermont | 2008 | NA | NA |
| State_Vermont | 2009 | NA | 0 |
| State_Vermont | 2010 | NA | NA |
| State_Vermont | 2011 | NA | NA |
| State_Vermont | 2012 | NA | 0 |
| State_Vermont | 2013 | NA | 0 |
| State_Vermont | 2014 | NA | 0 |
| State_Vermont | 2015 | NA | NA |
| State_Vermont | 2016 | NA | NA |
| State_Vermont | 2017 | NA | NA |
| State_Vermont | 2018 | NA | 0 |
| State_Vermont | 2019 | NA | NA |
| State_Vermont | 2020 | NA | NA |
| State_Vermont | 2021 | NA | NA |
| State_Vermont | 2022 | NA | 0 |
| State_Vermont | 2023 | NA | NA |
| State_Virginia | 1999 | NA | NA |
| State_Virginia | 2000 | NA | 0.094720271 |
| State_Virginia | 2001 | NA | NA |
| State_Virginia | 2002 | NA | 0.077003382 |
| State_Virginia | 2003 | NA | NA |
| State_Virginia | 2004 | NA | NA |
| State_Virginia | 2005 | NA | NA |
| State_Virginia | 2006 | NA | NA |
| State_Virginia | 2007 | NA | NA |
| State_Virginia | 2008 | NA | NA |
| State_Virginia | 2009 | NA | NA |
| State_Virginia | 2010 | NA | NA |
| State_Virginia | 2011 | NA | NA |
| State_Virginia | 2012 | NA | NA |
| State_Virginia | 2013 | NA | NA |
| State_Virginia | 2014 | NA | NA |
| State_Virginia | 2015 | NA | NA |
| State_Virginia | 2016 | NA | NA |
| State_Virginia | 2017 | NA | NA |
| State_Virginia | 2018 | NA | NA |
| State_Virginia | 2019 | NA | NA |
| State_Virginia | 2020 | NA | 0.049708414 |
| State_Virginia | 2021 | NA | NA |
| State_Virginia | 2022 | NA | 0.051744781 |
| State_Virginia | 2023 | NA | NA |
| State_Washington | 1999 | NA | NA |
| State_Washington | 2000 | NA | NA |
| State_Washington | 2001 | NA | NA |
| State_Washington | 2002 | NA | NA |
| State_Washington | 2003 | NA | NA |
| State_Washington | 2004 | NA | NA |
| State_Washington | 2005 | NA | NA |
| State_Washington | 2006 | NA | NA |
| State_Washington | 2007 | NA | NA |
| State_Washington | 2008 | NA | NA |
| State_Washington | 2009 | NA | 0.078906443 |
| State_Washington | 2010 | NA | NA |
| State_Washington | 2011 | NA | NA |
| State_Washington | 2012 | NA | NA |
| State_Washington | 2013 | NA | NA |
| State_Washington | 2014 | NA | 0.06504231 |
| State_Washington | 2015 | NA | NA |
| State_Washington | 2016 | NA | NA |
| State_Washington | 2017 | NA | NA |
| State_Washington | 2018 | NA | 0.053850314 |
| State_Washington | 2019 | NA | NA |
| State_Washington | 2020 | NA | NA |
| State_Washington | 2021 | NA | 0.055799261 |
| State_Washington | 2022 | NA | 0.063141749 |
| State_Washington | 2023 | NA | NA |
| State_West Virginia | 1999 | NA | NA |
| State_West Virginia | 2000 | NA | NA |
| State_West Virginia | 2001 | NA | NA |
| State_West Virginia | 2002 | NA | NA |
| State_West Virginia | 2003 | NA | NA |
| State_West Virginia | 2004 | NA | NA |
| State_West Virginia | 2005 | NA | NA |
| State_West Virginia | 2006 | NA | NA |
| State_West Virginia | 2007 | NA | NA |
| State_West Virginia | 2008 | NA | NA |
| State_West Virginia | 2009 | NA | NA |
| State_West Virginia | 2010 | NA | NA |
| State_West Virginia | 2011 | NA | NA |
| State_West Virginia | 2012 | NA | NA |
| State_West Virginia | 2013 | NA | NA |
| State_West Virginia | 2014 | NA | NA |
| State_West Virginia | 2015 | NA | NA |
| State_West Virginia | 2016 | NA | NA |
| State_West Virginia | 2017 | NA | NA |
| State_West Virginia | 2018 | NA | 0 |
| State_West Virginia | 2019 | NA | 0 |
| State_West Virginia | 2020 | NA | NA |
| State_West Virginia | 2021 | NA | NA |
| State_West Virginia | 2022 | NA | 0 |
| State_West Virginia | 2023 | NA | NA |
| State_Wisconsin | 1999 | NA | NA |
| State_Wisconsin | 2000 | NA | NA |
| State_Wisconsin | 2001 | NA | NA |
| State_Wisconsin | 2002 | NA | NA |
| State_Wisconsin | 2003 | NA | NA |
| State_Wisconsin | 2004 | NA | NA |
| State_Wisconsin | 2005 | NA | NA |
| State_Wisconsin | 2006 | NA | NA |
| State_Wisconsin | 2007 | NA | NA |
| State_Wisconsin | 2008 | NA | NA |
| State_Wisconsin | 2009 | NA | NA |
| State_Wisconsin | 2010 | NA | NA |
| State_Wisconsin | 2011 | NA | NA |
| State_Wisconsin | 2012 | NA | NA |
| State_Wisconsin | 2013 | NA | NA |
| State_Wisconsin | 2014 | NA | NA |
| State_Wisconsin | 2015 | NA | 0.070292247 |
| State_Wisconsin | 2016 | NA | NA |
| State_Wisconsin | 2017 | NA | NA |
| State_Wisconsin | 2018 | NA | NA |
| State_Wisconsin | 2019 | NA | 0.059043964 |
| State_Wisconsin | 2020 | NA | NA |
| State_Wisconsin | 2021 | NA | NA |
| State_Wisconsin | 2022 | NA | NA |
| State_Wisconsin | 2023 | NA | NA |
| State_Wyoming | 1999 | NA | NA |
| State_Wyoming | 2000 | NA | NA |
| State_Wyoming | 2001 | NA | 0 |
| State_Wyoming | 2002 | NA | 0 |
| State_Wyoming | 2003 | NA | 0 |
| State_Wyoming | 2004 | NA | 0 |
| State_Wyoming | 2005 | NA | 0 |
| State_Wyoming | 2006 | NA | 0 |
| State_Wyoming | 2007 | NA | NA |
| State_Wyoming | 2008 | NA | 0 |
| State_Wyoming | 2009 | NA | 0 |
| State_Wyoming | 2010 | NA | NA |
| State_Wyoming | 2011 | NA | NA |
| State_Wyoming | 2012 | NA | 0 |
| State_Wyoming | 2013 | NA | NA |
| State_Wyoming | 2014 | NA | 0 |
| State_Wyoming | 2015 | NA | 0 |
| State_Wyoming | 2016 | NA | NA |
| State_Wyoming | 2017 | NA | 0 |
| State_Wyoming | 2018 | NA | 0 |
| State_Wyoming | 2019 | NA | NA |
| State_Wyoming | 2020 | NA | NA |
| State_Wyoming | 2021 | NA | NA |
| State_Wyoming | 2022 | NA | NA |
| State_Wyoming | 2023 | NA | 0 |
| Urbanization_Metropolitan | 1999 | 0.129162559 | 0.009559801 |
| Urbanization_Metropolitan | 2000 | 0.133437988 | 0.00961142 |
| Urbanization_Metropolitan | 2001 | 0.128641453 | 0.009528783 |
| Urbanization_Metropolitan | 2002 | 0.107578154 | 0.00860447 |
| Urbanization_Metropolitan | 2003 | 0.104300606 | 0.008050584 |
| Urbanization_Metropolitan | 2004 | 0.114512403 | 0.008739994 |
| Urbanization_Metropolitan | 2005 | 0.104300606 | 0.007894308 |
| Urbanization_Metropolitan | 2006 | 0.106698873 | 0.00807166 |
| Urbanization_Metropolitan | 2007 | 0.117792919 | 0.00852606 |
| Urbanization_Metropolitan | 2008 | 0.105179887 | 0.008088782 |
| Urbanization_Metropolitan | 2009 | 0.119308937 | 0.008574135 |
| Urbanization_Metropolitan | 2010 | 0.114512403 | 0.007997975 |
| Urbanization_Metropolitan | 2011 | 0.104300606 | 0.007455287 |
| Urbanization_Metropolitan | 2012 | 0.099504071 | 0.007354889 |
| Urbanization_Metropolitan | 2013 | 0.100643906 | 0.007247882 |
| Urbanization_Metropolitan | 2014 | 0.092569823 | 0.006934688 |
| Urbanization_Metropolitan | 2015 | 0.090171556 | 0.006685783 |
| Urbanization_Metropolitan | 2016 | 0.08083904 | 0.005989065 |
| Urbanization_Metropolitan | 2017 | 0.09496809 | 0.006852627 |
| Urbanization_Metropolitan | 2018 | 0.121494949 | 0.00799543 |
| Urbanization_Metropolitan | 2019 | 0.088033841 | 0.006201532 |
| Urbanization_Metropolitan | 2020 | 0.099504071 | 0.006529939 |
| Urbanization_Nonmetropolitan | 1999 | 0.169770172 | 0.022335828 |
| Urbanization_Nonmetropolitan | 2000 | 0.184781471 | 0.023328378 |
| Urbanization_Nonmetropolitan | 2001 | 0.126127555 | 0.019788891 |
| Urbanization_Nonmetropolitan | 2002 | 0.14768267 | 0.020497688 |
| Urbanization_Nonmetropolitan | 2003 | 0.149965306 | 0.020836214 |
| Urbanization_Nonmetropolitan | 2004 | 0.186658632 | 0.023075558 |
| Urbanization_Nonmetropolitan | 2005 | 0.155264937 | 0.020600019 |
| Urbanization_Nonmetropolitan | 2006 | 0.144550043 | 0.019680061 |
| Urbanization_Nonmetropolitan | 2007 | 0.171907886 | 0.021712715 |
| Urbanization_Nonmetropolitan | 2008 | 0.122705085 | 0.018497508 |
| Urbanization_Nonmetropolitan | 2009 | 0.1283809 | 0.018390954 |
| Urbanization_Nonmetropolitan | 2010 | 0.153652294 | 0.020560462 |
| Urbanization_Nonmetropolitan | 2011 | 0.175261014 | 0.024681931 |
| Urbanization_Nonmetropolitan | 2012 | 0.136718504 | 0.018525733 |
| Urbanization_Nonmetropolitan | 2013 | 0.095586818 | 0.015727358 |
| Urbanization_Nonmetropolitan | 2014 | 0.124987721 | 0.018151711 |
| Urbanization_Nonmetropolitan | 2015 | 0.158825013 | 0.021571989 |
| Urbanization_Nonmetropolitan | 2016 | 0.123844919 | 0.017222082 |
| Urbanization_Nonmetropolitan | 2017 | 0.122065379 | 0.017008463 |
| Urbanization_Nonmetropolitan | 2018 | 0.119357235 | 0.016511002 |
| Urbanization_Nonmetropolitan | 2019 | 0.166899095 | 0.019972942 |
| Urbanization_Nonmetropolitan | 2020 | 0.17345716 | 0.020903988 |
| Age Groups_25-34 years | 1999 | NA | NA |
| Age Groups_25-34 years | 2000 | NA | NA |
| Age Groups_25-34 years | 2001 | NA | 0 |
| Age Groups_25-34 years | 2002 | NA | NA |
| Age Groups_25-34 years | 2003 | NA | 0 |
| Age Groups_25-34 years | 2004 | NA | NA |
| Age Groups_25-34 years | 2005 | NA | 0 |
| Age Groups_25-34 years | 2006 | NA | 0 |
| Age Groups_25-34 years | 2007 | NA | 0 |
| Age Groups_25-34 years | 2008 | NA | NA |
| Age Groups_25-34 years | 2009 | NA | 0 |
| Age Groups_25-34 years | 2010 | NA | NA |
| Age Groups_25-34 years | 2011 | NA | 0 |
| Age Groups_25-34 years | 2012 | NA | 0 |
| Age Groups_25-34 years | 2013 | NA | 0 |
| Age Groups_25-34 years | 2014 | NA | NA |
| Age Groups_25-34 years | 2015 | NA | NA |
| Age Groups_25-34 years | 2016 | NA | 0 |
| Age Groups_25-34 years | 2017 | NA | 0 |
| Age Groups_25-34 years | 2018 | NA | NA |
| Age Groups_25-34 years | 2019 | NA | 0 |
| Age Groups_25-34 years | 2020 | NA | NA |
| Age Groups_25-34 years | 2021 | NA | 0 |
| Age Groups_25-34 years | 2022 | NA | 0 |
| Age Groups_25-34 years | 2023 | NA | NA |
| Age Groups_35-44 years | 1999 | NA | NA |
| Age Groups_35-44 years | 2000 | NA | NA |
| Age Groups_35-44 years | 2001 | NA | NA |
| Age Groups_35-44 years | 2002 | NA | NA |
| Age Groups_35-44 years | 2003 | NA | NA |
| Age Groups_35-44 years | 2004 | NA | NA |
| Age Groups_35-44 years | 2005 | NA | NA |
| Age Groups_35-44 years | 2006 | NA | NA |
| Age Groups_35-44 years | 2007 | NA | NA |
| Age Groups_35-44 years | 2008 | NA | NA |
| Age Groups_35-44 years | 2009 | NA | NA |
| Age Groups_35-44 years | 2010 | NA | NA |
| Age Groups_35-44 years | 2011 | NA | NA |
| Age Groups_35-44 years | 2012 | NA | NA |
| Age Groups_35-44 years | 2013 | NA | NA |
| Age Groups_35-44 years | 2014 | NA | NA |
| Age Groups_35-44 years | 2015 | NA | NA |
| Age Groups_35-44 years | 2016 | NA | NA |
| Age Groups_35-44 years | 2017 | NA | NA |
| Age Groups_35-44 years | 2018 | NA | NA |
| Age Groups_35-44 years | 2019 | NA | NA |
| Age Groups_35-44 years | 2020 | NA | NA |
| Age Groups_35-44 years | 2021 | NA | NA |
| Age Groups_35-44 years | 2022 | NA | 0.008563 |
| Age Groups_35-44 years | 2023 | NA | NA |
| Age Groups_45-54 years | 1999 | NA | NA |
| Age Groups_45-54 years | 2000 | NA | 0.008803 |
| Age Groups_45-54 years | 2001 | NA | 0.009833 |
| Age Groups_45-54 years | 2002 | NA | NA |
| Age Groups_45-54 years | 2003 | NA | 0.008833 |
| Age Groups_45-54 years | 2004 | NA | 0.009608 |
| Age Groups_45-54 years | 2005 | NA | 0.009702 |
| Age Groups_45-54 years | 2006 | NA | 0.009525 |
| Age Groups_45-54 years | 2007 | 0.045517 | 0.010178 |
| Age Groups_45-54 years | 2008 | NA | 0.008416 |
| Age Groups_45-54 years | 2009 | NA | 0.00919 |
| Age Groups_45-54 years | 2010 | 0.053325 | 0.010885 |
| Age Groups_45-54 years | 2011 | 0.058142 | 0.011403 |
| Age Groups_45-54 years | 2012 | NA | 0.008145 |
| Age Groups_45-54 years | 2013 | NA | 0.008238 |
| Age Groups_45-54 years | 2014 | NA | 0.009487 |
| Age Groups_45-54 years | 2015 | NA | 0.009547 |
| Age Groups_45-54 years | 2016 | NA | 0.007391 |
| Age Groups_45-54 years | 2017 | NA | 0.010286 |
| Age Groups_45-54 years | 2018 | 0.069658 | 0.012935 |
| Age Groups_45-54 years | 2019 | NA | 0.008821 |
| Age Groups_45-54 years | 2020 | NA | 0.009595 |
| Age Groups_45-54 years | 2021 | NA | 0.010133 |
| Age Groups_45-54 years | 2022 | 0.076673 | 0.013771 |
| Age Groups_45-54 years | 2023 | 0.071616 | 0.013299 |
| Age Groups_55-64 years | 1999 | 0.096728 | 0.020169 |
| Age Groups_55-64 years | 2000 | 0.127705 | 0.022937 |
| Age Groups_55-64 years | 2001 | 0.131446 | 0.022882 |
| Age Groups_55-64 years | 2002 | 0.112346 | 0.020511 |
| Age Groups_55-64 years | 2003 | 0.107109 | 0.019555 |
| Age Groups_55-64 years | 2004 | 0.112608 | 0.019602 |
| Age Groups_55-64 years | 2005 | 0.114224 | 0.019307 |
| Age Groups_55-64 years | 2006 | 0.087692 | 0.016572 |
| Age Groups_55-64 years | 2007 | 0.144891 | 0.020913 |
| Age Groups_55-64 years | 2008 | 0.111251 | 0.018047 |
| Age Groups_55-64 years | 2009 | 0.090381 | 0.015977 |
| Age Groups_55-64 years | 2010 | 0.1069 | 0.017118 |
| Age Groups_55-64 years | 2011 | 0.144501 | 0.019484 |
| Age Groups_55-64 years | 2012 | 0.126988 | 0.018141 |
| Age Groups_55-64 years | 2013 | 0.094108 | 0.015471 |
| Age Groups_55-64 years | 2014 | 0.114777 | 0.016923 |
| Age Groups_55-64 years | 2015 | 0.088067 | 0.014678 |
| Age Groups_55-64 years | 2016 | 0.118177 | 0.016882 |
| Age Groups_55-64 years | 2017 | 0.116679 | 0.016668 |
| Age Groups_55-64 years | 2018 | 0.137205 | 0.018016 |
| Age Groups_55-64 years | 2019 | 0.087164 | 0.01433 |
| Age Groups_55-64 years | 2020 | 0.117914 | 0.016676 |
| Age Groups_55-64 years | 2021 | 0.095788 | 0.01496 |
| Age Groups_55-64 years | 2022 | 0.137815 | 0.018096 |
| Age Groups_55-64 years | 2023 | 0.172025 | 0.020273 |
| Age Groups_65-74 years | 1999 | 0.314894 | 0.041348 |
| Age Groups_65-74 years | 2000 | 0.271872 | 0.038449 |
| Age Groups_65-74 years | 2001 | 0.266534 | 0.038076 |
| Age Groups_65-74 years | 2002 | 0.261032 | 0.037677 |
| Age Groups_65-74 years | 2003 | 0.243231 | 0.036259 |
| Age Groups_65-74 years | 2004 | 0.283915 | 0.038999 |
| Age Groups_65-74 years | 2005 | 0.270103 | 0.037822 |
| Age Groups_65-74 years | 2006 | 0.239546 | 0.035319 |
| Age Groups_65-74 years | 2007 | 0.263976 | 0.036607 |
| Age Groups_65-74 years | 2008 | 0.248712 | 0.034827 |
| Age Groups_65-74 years | 2009 | 0.273158 | 0.035867 |
| Age Groups_65-74 years | 2010 | 0.317776 | 0.038256 |
| Age Groups_65-74 years | 2011 | 0.240195 | 0.032686 |
| Age Groups_65-74 years | 2012 | 0.229306 | 0.03092 |
| Age Groups_65-74 years | 2013 | 0.269662 | 0.032701 |
| Age Groups_65-74 years | 2014 | 0.234864 | 0.029828 |
| Age Groups_65-74 years | 2015 | 0.257708 | 0.030584 |
| Age Groups_65-74 years | 2016 | 0.230525 | 0.028376 |
| Age Groups_65-74 years | 2017 | 0.222346 | 0.027369 |
| Age Groups_65-74 years | 2018 | 0.291877 | 0.030939 |
| Age Groups_65-74 years | 2019 | 0.241397 | 0.02769 |
| Age Groups_65-74 years | 2020 | 0.230419 | 0.026606 |
| Age Groups_65-74 years | 2021 | 0.34159 | 0.031853 |
| Age Groups_65-74 years | 2022 | 0.319636 | 0.030757 |
| Age Groups_65-74 years | 2023 | 0.296956 | 0.02926 |
| Age Groups_75-84 years | 1999 | 0.580781 | 0.068926 |
| Age Groups_75-84 years | 2000 | 0.776625 | 0.079264 |
| Age Groups_75-84 years | 2001 | 0.60348 | 0.069224 |
| Age Groups_75-84 years | 2002 | 0.618886 | 0.06963 |
| Age Groups_75-84 years | 2003 | 0.659097 | 0.071489 |
| Age Groups_75-84 years | 2004 | 0.677449 | 0.072216 |
| Age Groups_75-84 years | 2005 | 0.619512 | 0.068835 |
| Age Groups_75-84 years | 2006 | 0.626186 | 0.069151 |
| Age Groups_75-84 years | 2007 | 0.649478 | 0.070446 |
| Age Groups_75-84 years | 2008 | 0.55827 | 0.065341 |
| Age Groups_75-84 years | 2009 | 0.637345 | 0.069958 |
| Age Groups_75-84 years | 2010 | 0.627817 | 0.069331 |
| Age Groups_75-84 years | 2011 | 0.62997 | 0.069148 |
| Age Groups_75-84 years | 2012 | 0.602744 | 0.067389 |
| Age Groups_75-84 years | 2013 | 0.438775 | 0.057124 |
| Age Groups_75-84 years | 2014 | 0.475053 | 0.058923 |
| Age Groups_75-84 years | 2015 | 0.531488 | 0.061784 |
| Age Groups_75-84 years | 2016 | 0.484771 | 0.05836 |
| Age Groups_75-84 years | 2017 | 0.489578 | 0.057697 |
| Age Groups_75-84 years | 2018 | 0.383257 | 0.049896 |
| Age Groups_75-84 years | 2019 | 0.48842 | 0.055303 |
| Age Groups_75-84 years | 2020 | 0.620002 | 0.061389 |
| Age Groups_75-84 years | 2021 | 0.746634 | 0.067876 |
| Age Groups_75-84 years | 2022 | 0.667787 | 0.061737 |
| Age Groups_75-84 years | 2023 | 0.642418 | 0.059139 |
| Age Groups_85+ years | 1999 | 2.142504 | 0.227105 |
| Age Groups_85+ years | 2000 | 1.886976 | 0.21097 |
| Age Groups_85+ years | 2001 | 1.553625 | 0.189806 |
| Age Groups_85+ years | 2002 | 1.304704 | 0.172812 |
| Age Groups_85+ years | 2003 | 1.365822 | 0.174876 |
| Age Groups_85+ years | 2004 | 1.407867 | 0.175983 |
| Age Groups_85+ years | 2005 | 1.384953 | 0.171782 |
| Age Groups_85+ years | 2006 | 1.520778 | 0.176787 |
| Age Groups_85+ years | 2007 | 1.389014 | 0.166019 |
| Age Groups_85+ years | 2008 | 1.077785 | 0.144025 |
| Age Groups_85+ years | 2009 | 1.341456 | 0.158092 |
| Age Groups_85+ years | 2010 | 1.274249 | 0.152302 |
| Age Groups_85+ years | 2011 | 1.167823 | 0.142672 |
| Age Groups_85+ years | 2012 | 1.08708 | 0.135885 |
| Age Groups_85+ years | 2013 | 1.142235 | 0.137509 |
| Age Groups_85+ years | 2014 | 1.054813 | 0.130833 |
| Age Groups_85+ years | 2015 | 1.033853 | 0.128234 |
| Age Groups_85+ years | 2016 | 0.893371 | 0.11833 |
| Age Groups_85+ years | 2017 | 1.066678 | 0.128413 |
| Age Groups_85+ years | 2018 | 1.23768 | 0.13752 |
| Age Groups_85+ years | 2019 | 1.196071 | 0.134569 |
| Age Groups_85+ years | 2020 | 1.156431 | 0.131787 |
| Age Groups_85+ years | 2021 | 1.472617 | 0.156982 |
| Age Groups_85+ years | 2022 | 1.557232 | 0.15495 |
| Age Groups_85+ years | 2023 | 1.485073 | 0.15483 |

Supplementary table 2. Characteristics with APC, 95% CI, and Time Range.

| Metric | Measure | Start | End | APC | lower | upper | P value |
| --- | --- | --- | --- | --- | --- | --- | --- |
| Census Region | Midwest | 1999 | 2023 | -1.8714 | -2.667 | -1.0694 | 0.000076 |
| Census Region | Northeast | 1999 | 2023 | -0.0867 | -1.154 | 0.9921 | 0.868789 |
| Census Region | South | 1999 | 2017 | -2.2137 | -3.0023 | -1.4187 | 0.000012 |
| Census Region | South | 2017 | 2023 | 10.9905 | 7.2389 | 14.8733 | 0.000004 |
| Census Region | West | 1999 | 2023 | 1.565 | 0.5047 | 2.6365 | 0.005537 |
| Race | NH Black | 1999 | 2023 | -0.53 | -1.7246 | 0.6792 | 0.372386 |
| Race | NH White | 1999 | 2016 | -2.1738 | -3.2465 | -1.0891 | 0.000487 |
| Race | NH White | 2016 | 2023 | 7.3239 | 3.2551 | 11.553 | 0.001084 |
| Sex | Both | 1999 | 2019 | -1.5035 | -2.3139 | -0.6863 | 0.00106 |
| Sex | Both | 2019 | 2023 | 13.5469 | 4.7802 | 23.0472 | 0.003592 |
| Sex | Female | 1999 | 2017 | -2.7464 | -3.8017 | -1.6794 | 0.000033 |
| Sex | Female | 2017 | 2023 | 7.5896 | 2.1795 | 13.2862 | 0.007782 |
| Sex | Male | 1999 | 2019 | -0.1395 | -1.1115 | 0.8421 | 0.769021 |
| Sex | Male | 2019 | 2023 | 8.6635 | -0.2745 | 18.4026 | 0.057078 |
| State | California | 1999 | 2023 | 0.2828 | -0.757 | 1.3335 | 0.580521 |
| Urbanization | Metropolitan | 1999 | 2020 | -1.329 | -2.0137 | -0.6395 | 0.000691 |
| Urbanization | Nonmetropolitan | 1999 | 2020 | -0.7221 | -1.8326 | 0.401 | 0.194053 |
| Age Groups | 55-64 years | 1999 | 2023 | 0.6232 | -0.4609 | 1.719 | 0.247564 |
| Age Groups | 65-74 years | 1999 | 2017 | -0.8512 | -1.9416 | 0.2513 | 0.122511 |
| Age Groups | 65-74 years | 2017 | 2023 | 4.8285 | 0.1479 | 9.7279 | 0.043664 |
| Age Groups | 75-84 years | 1999 | 2011 | -0.7442 | -2.3762 | 0.9152 | 0.350317 |
| Age Groups | 75-84 years | 2011 | 2018 | -5.1836 | -9.7974 | -0.3338 | 0.03817 |
| Age Groups | 75-84 years | 2018 | 2021 | 20.8826 | -7.5067 | 57.9855 | 0.150883 |
| Age Groups | 75-84 years | 2021 | 2023 | -7.3011 | -26.5083 | 16.9259 | 0.495199 |
| Age Groups | 85+ years | 1999 | 2001 | -15.8151 | -32.2377 | 4.5876 | 0.112482 |
| Age Groups | 85+ years | 2001 | 2016 | -2.7705 | -3.9028 | -1.6249 | 0.000097 |
| Age Groups | 85+ years | 2016 | 2023 | 6.4651 | 3.3969 | 9.6243 | 0.000303 |

Supplementary table 3. Characteristics with AAPC, 95% CI, and Time Range.

| Metric | Measure | Start | End | APC | lower | upper | P value |
| --- | --- | --- | --- | --- | --- | --- | --- |
| Census Region | Midwest | -1.8714 | -2.667 | -1.0694 | 0.000076 | Census Region | Midwest |
| Census Region | Northeast | -0.0867 | -1.154 | 0.9921 | 0.868789 | Census Region | Northeast |
| Census Region | South | 0.9322 | -0.061 | 1.9354 | 0.065913 | Census Region | South |
| Census Region | West | 1.565 | 0.5047 | 2.6365 | 0.005537 | Census Region | West |
| Race | NH Black | -0.53 | -1.7246 | 0.6792 | 0.372386 | Race | NH Black |
| Race | NH White | 0.5061 | -0.7807 | 1.8095 | 0.442595 | Race | NH White |
| Sex | Both | 0.8587 | -0.5583 | 2.2959 | 0.236242 | Sex | Both |
| Sex | Female | -0.2594 | -1.6807 | 1.1824 | 0.72281 | Sex | Female |
| Sex | Male | 1.2765 | -0.2782 | 2.8555 | 0.108059 | Sex | Male |
| State | California | 0.2828 | -0.757 | 1.3335 | 0.580521 | State | California |
| Urbanization | Metropolitan | -1.329 | -2.0137 | -0.6395 | 0.000691 | Urbanization | Metropolitan |
| Urbanization | Nonmetropolitan | -0.7221 | -1.8326 | 0.401 | 0.194053 | Urbanization | Nonmetropolitan |
| Age Groups | 55-64 years | 0.6232 | -0.4609 | 1.719 | 0.247564 | Age Groups | 55-64 years |
| Age Groups | 65-74 years | 0.5392 | -0.7852 | 1.8813 | 0.426735 | Age Groups | 65-74 years |
| Age Groups | 75-84 years | -0.1869 | -3.9561 | 3.7301 | 0.924104 | Age Groups | 75-84 years |
| Age Groups | 85+ years | -1.3542 | -3.2862 | 0.6165 | 0.1767 | Age Groups | 85+ years |
